# CircRNA-3302 promotes endothelial-to-mesenchymal transition via sponging miR-135b-5p to enhance KIT expression in Kawasaki disease

**DOI:** 10.1038/s41420-022-01092-4

**Published:** 2022-06-29

**Authors:** Chao Ni, Huixian Qiu, Shuchi Zhang, Qihao Zhang, Ruiyin Zhang, Jinhui Zhou, Jinshun Zhu, Chao Niu, Rongzhou Wu, Chuxiao Shao, Abdullah Al Mamun, Bo Han, Maoping Chu, Chang Jia

**Affiliations:** 1 Pediatric Research Institute, The Second Affiliated Hospital and Yuying Children’s Hospital of Wenzhou Medical University, 325027 Wenzhou, China; 2 Key Laboratory of Structural Malformations in Childern of Zhejiang Province, 325027 Wenzhou, China; 3 Children’s Heart Center, The Second Affiliated Hospital and Yuying Children’s Hospital of Wenzhou Medical University, 325027 Wenzhou, China; 4 Department of Hepatopancreatobiliary Surgery, Lishui Central Hospital, The Fifth Affiliated Hospital of Wenzhou Medical University, Lishui Hospital of Zhejiang University, Lishui, 323000 Zhejiang China; 5 Molecular Pharmacology Research Center, School of Pharmaceutical Sciences, Wenzhou Medical University, Wenzhou, 325035 Zhejiang Province China; 6 Department of Pediatric Cardiology, Shandong Provincial Hospital Affiliated to Shandong First Medical University, Jinan, Shandong 250021 China

**Keywords:** Mechanisms of disease, Vascular diseases

## Abstract

Endothelial-to-mesenchymal transition (EndMT) is implicated in myofibroblast-like cell-mediated damage to coronary artery wall of Kawasaki disease (KD) patients, which subsequently increases the risk of coronary artery aneurysm. Many circular RNAs (circRNAs) have been reported to be associated with cardiovascular diseases. However, the roles and underlying molecular mechanism of circRNAs in KD-associated EndMT remains indefinite. In this research, we screened out circRNA-3302 from human umbilical vein endothelial cells (HUVECs) treated by sera from healthy controls (HCs) or KD patients via circRNA sequencing (circRNA-seq). In addition, circRNA-3302 upregulation was verified in endothelial cells stimulated by KD serum and pathological KD mice modeled with *Candida albicans* cell wall extracts (CAWS). Moreover, in vitro experiments demonstrated that overexpression of circRNA-3302 could markedly induce EndMT, and silencing of circRNA-3302 significantly alleviated KD serum-mediated EndMT. To further explore the molecular mechanisms of circRNA-3302 inducing EndMT, RNA sequencing (RNA-seq), a dual-luciferase reporter system, nuclear and extra-nuclear RNA isolation, RT-qPCR and Western blot analyses and so on, were utilized. Our data demonstrated that circRNA-3302 contributed to the KD-associated EndMT via sponging miR-135b-5p to enhance KIT expression. Collectively, our results imply that circRNA-3302 plays an important role in KD-associated EndMT, providing new insights into minimizing the risks of developing coronary artery aneurysms.

## Introduction

Kawasaki disease (KD) is an acute self-limiting vasculitis of unknown etiology, which is mainly found in 5-year-old children [1]. KD has been reported as the leading cause of the acquired heart disease in developed countries, which may further lead to coronary artery aneurysms (CAA) among 20–25% of untreated patients and even in 5% population of patients who received therapy with IVIG [2]. Classically presented with lasting fever, strawberry tongue, peeling of hands and feet, palm and soles erythema, KD has long been suspected with an infectious etiology, but the etiopathology remains unknown [1, 3]. IVIG plus aspirin is the current effective therapeutic option for the treatment and management of KD, contributing to alleviating systemic inflammation and protecting from CAA formation [4]. However, IVIG-resistant patients are still at high risk to develop coronary lesions and require other effective therapeutic strategies [1, 5]. Though cutting-edged imaging advancement and novel strategies for suppressing inflammation have contributed to clinical diagnosis and treatment, the primary knowledge of molecular mechanisms in aneurysm formation during KD pathophysiology has not been clearly understood [6]. Thus, more pre-clinical research is highly warranted to explore potential therapeutic agents for the regulation of early-stage aneurysms.

Endothelial-to-mesenchymal transition (EndMT) is a potential molecular mechanism in response to injuries where endothelial cells diminish their endothelial-specific markers and convert into mesenchymal phenotypes [7]. EndMT is a dynamic mechanism involved in heart development, fibrosis, diabetic renal disease and cancer [8]. During this complicated cell-lineage transition, the downregulation of endothelial cell adhesion molecules, including VE-cadherin, ZO-1 and Claudin-1, and the upregulation of mesenchymal markers, including Vimentin, α-SMA and β-catenin [9, 10], lead to a massive loss of adhesion and reconstruction of the cytoskeleton in endothelial cells, finally resulting in a striking morphologic change [11] and acquisition of invasive and migratory properties [12]. This fate-lineage transition induces an inflammatory response and damages the vascular wall [13]. KD autopsies uncovered the presence of myofibroblast-like cells infiltrated into the artery walls, and these myofibroblast-like cells are responsible for aneurysm formation via secreting IL-17, promoting the release of IL-6, granulocyte-macrophage colony-stimulating factor (GM-CSF), and vascular endothelial growth factor (VEGF), and recruitment of neutrophils, CD8^+^T cells and CD86^+^ M1 macrophages [14]. Previous studies implicated that these myofibroblast-like cells may derive from endothelial cells via EndMT [6, 15], indicating that EndMT is a pivotal contributor to the pathogenesis of KD.

Circular RNAs (circRNAs), a newly-identified class of non-coding RNAs with 3′ and 5′ ends covalently bonded, are conservative among different species [16]. Initially, circRNA was serendipitously discovered and reported as a byproduct of abnormal RNA splicing or splicing errors [17]. With the development of high-throughput sequencing, thousands of circRNAs have been successfully identified [18, 19]. In recent years, circRNAs have been reported to be widely expressed in tissue-specific environments, and its dysregulation may be associated with cardiovascular diseases, including cardiac fibrosis, cardiac senescence, cardiomyopathy, cardiac hypertrophy, myocardial infarction and heart failure, atherosclerosis, coronary artery disease and aneurysm [20–23]. Vausort et al. reported that Myocardial Infarction‐Associated CircRNA (MICA) is downregulated in the peripheral blood samples of MI patients, which may be used as a predictor of left ventricular dysfunction development in MI [24]. CircRNA‐010567 and circRNA‐000203 are closely related to diabetic myocardial fibrosis, which may provide a promising therapeutic approach [25, 26]. Zheng et al. found that circRNA 000595 is highly expressed in the aortic aneurysm tissue of patients with aortic aneurysm [27]. However, the precise roles and underlying mechanisms of circRNAs have not been reported in KD.

In this study, we aimed to investigate the significant role and the molecular mechanisms of circRNAs in the pathogenesis of KD. We first found that circ3302 was upregulated in both in vitro cell models and in vivo pathological models of KD. We then performed in vitro experiments to explore the molecular mechanism of circ3302 mediating EndMT in KD. Our results demonstrated that circ3302 absorbed miR-135b-5p to reduce its effect on KIT translation, thereby up-regulating the expression of KIT protein and facilitating the formation of EndMT. These findings provide clinical implications of circRNAs as a potential biomarker and therapeutic target for KD.

## Results

### EndMT existed in KD serum-treated HUVECs and *Candida albicans* cell wall extracts (CAWS)-induced KD mouse model

**Fig. 1:**
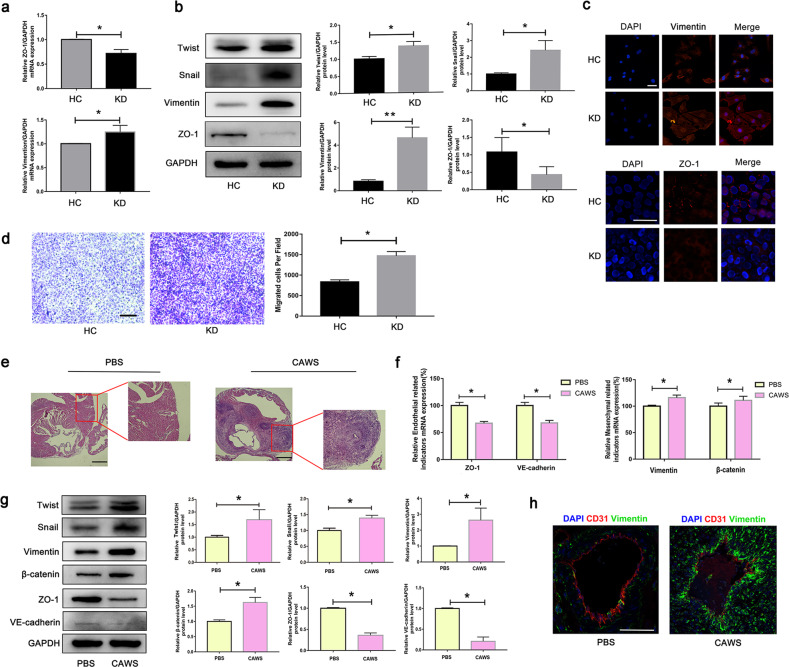
EndMT existed in both in vitro and in vivo KD models. **a–c** HUVECs were treated with medium containing 15% sera from health control (HC) (*n* = 6) or KD subjects (KD) (*n* = 6). After 48 h, the treated endothelial cells were harvested. ZO-1 and Vimentin mRNA levels were measured by RT-qPCR analysis (**a**). The protein levels of Twist, Snail, Vimentin and ZO-1 were examined by western blotting (**b**). The expression of Vimentin and ZO-1 were analyzed by immunofluorescence staining (**c**). Magnification: ×400 for Vimentin staining, and magnification:×1000 for ZO-1 staining. Scale bar = 50 μm for **c**. **d** Migration ability was measured by transwell test. Magnification: ×40. Scale bar = 100 μm. **e** After the mice were injected with PBS or CAWS, they were fed normally for 6 weeks, and the heart aorta root sections were obtained for HE staining. Magnification: ×40. Scale bar = 100 μm. **f–g** The heart tissues were harvested, and the total RNA and protein were respectively isolated for determining the expression of endothelial and mesenchymal markers. **h** Vimentin expression was analyzed in coronary endothelial cells using Vimentin/CD31 double staining. Magnification:×400. Scale bar = 50 μm. *P < 0.05 and **P < 0.01 vs. the HC group or PBS group.

To replicate EndMT in vitro and characterize the underlying molecular mechanism, human umbilical vein endothelial cells (HUVECs) were subjected to KD serum for 48–72 h. Results showed that EndMT was induced in KD-treated cells, as evidenced by the decreased mRNA level of the endothelial marker ZO-1 and the increased mRNA level of mesenchymal marker Vimentin (Fig. 1a), and the elevated protein levels of Twist, Snail and Vimentin, and declined protein expression of ZO-1 (Fig. 1b). The consistent expression changes of Vimentin and ZO-1 were also substantiated by immunofluorescence staining (Fig. 1c). As is known, EndoMT-derived cells gain a migration capacity, allowing them to reach the surrounding tissues [28]. Therefore, we also assessed the effect of KD serum on the migratory capacities of HUVECs. As anticipated, KD serum-treated endothelial cells exhibited a significant increase in migratory capacity (Fig. 1d). What’s more, similar phenotypes were also observed in CAWS-induced KD mice model, manifesting decreased ZO-1 and VE-cadherin expression, increased Vimentin and β-catenin expression, and elevated Twist and Snail expression (Fig. 1e–g). Moreover, immunofluorescence assay showed that CD31 expression was downregulated and Vimentin was upregulated in the coronary artery endothelium of CAWS-induced KD mice compared with PBS-treated mice (Fig. 1h). Together, these results confirm the induction of an EndMT-like profile in both in vitro and in vivo experiments.

### Characterize transcriptional profile and screen for potential circRNAs during EndMT in an in vitro model of KD

**Fig. 2:**
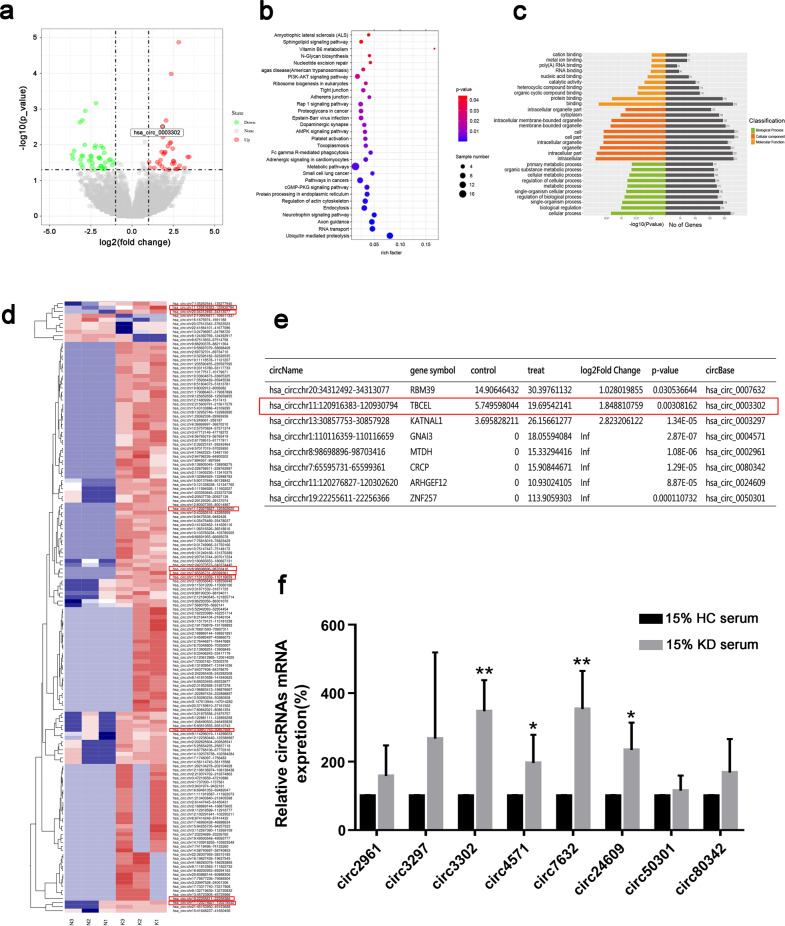
Sequencing results of circRNAs in KD-related EndoMT in vitro model and screening of candidate genes. **a** CircRNA expression profiling (Volcano plot) of HUVECs treated with 15% KD serum versus 15% HC serum (green represented the downregulated circRNAs and red represented the upregulated circRNAs). **b** KEGG pathway analysis of upregulated circRNAs in HUVECs treated with 15% KD serum when compared with 15% HC serum. The size of the dot represented the gene number included in the biological pathway, and the color represented *P* value. **c** Gene Ontology (GO) analysis of upregulated circRNAs in HUVECs treated with 15% KD serum when compared with 15% HC serum. **d** Heatmaps illustrated the expression of upregulated circRNAs. **e** Table of candidate significantly differential genes. **f** The expression of eight candidate circRNAs was confirmed by RT-qPCR analysis. Data were shown as mean ± SD (*n* = 3). *P < 0.05, and **P < 0.01 vs. the HC group.

To further distinguish the transcriptional profiles and explore the significant role of circRNAs in the EndMT model stimulated by KD serum, circRNA sequencing (circRNA-seq) was employed to characterize the transcriptional profile changes of circRNAs. We identified 279 differentially expressed circRNAs, with 51.97% of circRNAs upregulated and 48.03% downregulated (Fig. 2a). Next, we performed Gene Ontology (GO) and KEGG pathway analyses and found the upregulated circRNAs were associated with intercellular adhesion and junction (Fig. 2b, c). The genes with significant upregulation in the expression were exhibited as a heatmap (Fig. 2d). We selected eight circRNAs from these upregulated circRNAs (Fig. 2e). We further carried out RT-qPCR analyses to verify the differential expression patterns and eventually screened out four circRNAs: circ3302, circ4571, circ7632, and circ24609 (Fig. 2f). Considering the log_2_(KD/NC) and *P*-value shown in Fig. 2e, circ3302 was chosen to investigate the functions and molecular mechanism of circRNAs in the KD sera-induced EndMT model.

### Circ3302 upregulation induced EndMT

**Fig. 3:**
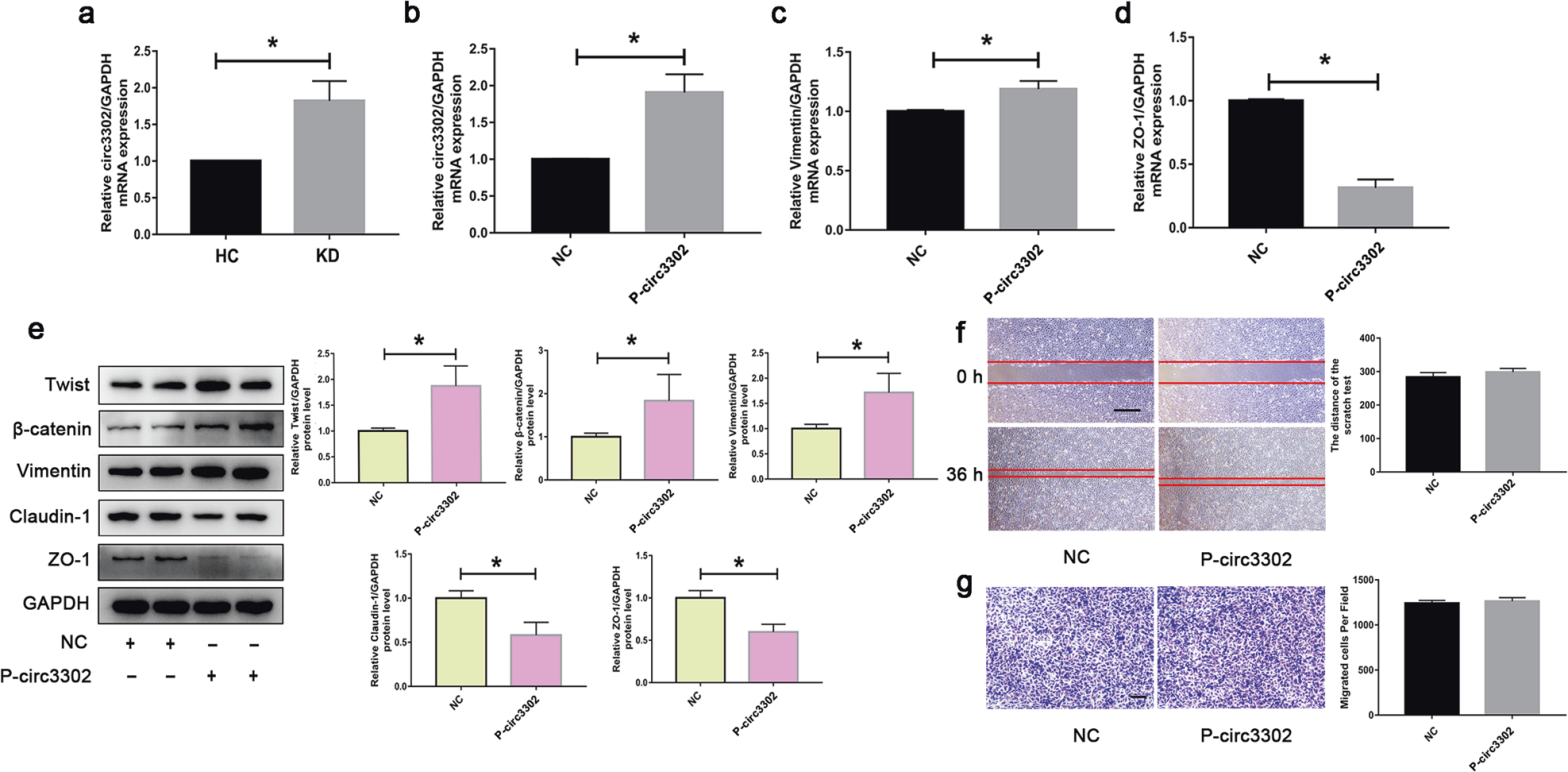
Upregulation of circ3302 induced EndMT. **a** HUVECs were treated with medium containing 15% sera from HC (*n* = 6) and KD subjects (*n* = 6). After 48 h, circ3302 levels were measured by RT-qPCR analysis. **b** HUVECs were transfected with Plasmid-circ3302 (P-circ3302) to overexpress circ3302. **c**, **d** The mRNA levels of Vimentin and ZO-1 were determined by RT-qPCR analysis. **e** The protein levels of Twist, β-catenin, Vimentin, Claudin-1 and ZO-1 were determined by Western blotting. **f**, **g** Effect of circ3302 overexpression on migration was respectively evaluated by scratch and transwell assays. Magnification:×40 and scale bar = 100 μm for scratch assay. Magnification:×100 and scale bar = 50 μm for transwell assay. Data were expressed as mean ± SD (*n* = 3). *P < 0.05 vs. the HC group or NC group.

To investigate the potential role of circ3302, its expression was again confirmed in HC and KD sera-treated endothelial cells. As shown in Fig. 3a, the expression of circ3302 was significantly elevated in KD-associated EndMT models. Moreover, the upregulation of circ3302 was also confirmed in CAWS-induced KD mice model (Fig. S1). To further explore whether circ3302 could induce EndMT, we overexpressed circ3302 in endothelial cells and observed its effect on the expression of EndMT-related proteins (Fig. 3b). As expected, overexpression of circ3302 significantly elevated Vimentin expression and decreased ZO-1 expression at the mRNA level (Fig. 3c, d), and upregulated Twist, β-catenin and Vimentin and downregulated Claudin-1 and ZO-1 at the protein level (Fig. 3e). However, scratch and transwell assays displayed that circ3302 overexpression had no effect on migration capacity (Fig.3f, g). Whereas, silencing of circ3302 led to the opposite expression change of the above-mentioned genes or proteins (Fig. S2). Taken together, these results indicated that upregulation of circ3302 could markedly induce EndMT.

### Loss of circ3302 alleviated KD-associated EndMT

**Fig. 4:**
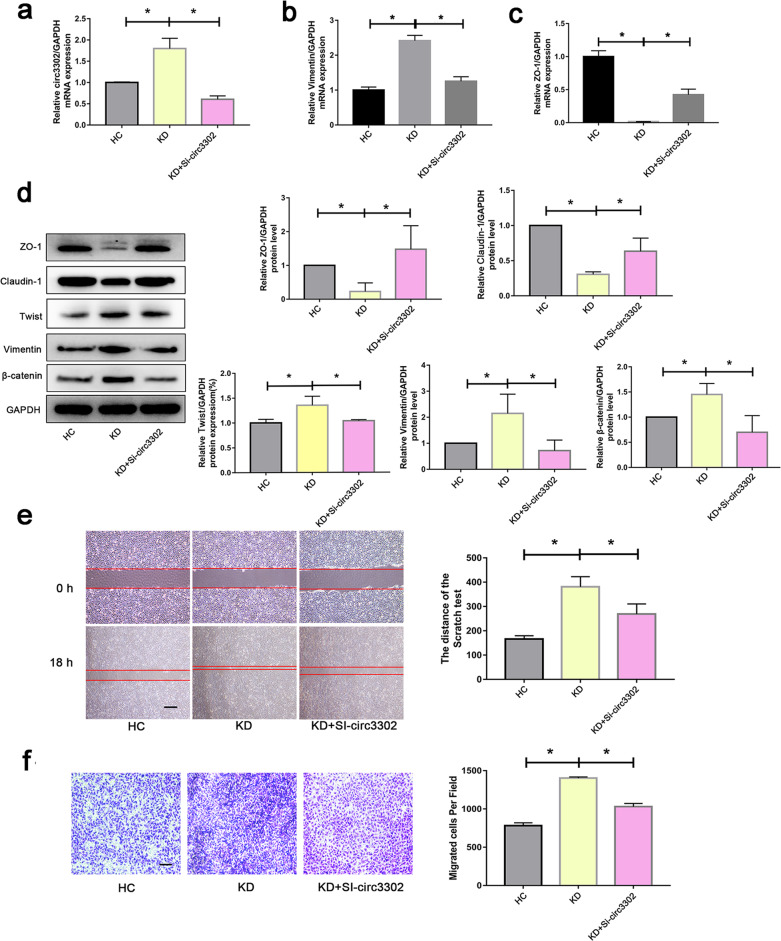
Effect of circ3302 silencing on KD serum-induced EndoMT. **a–c** HUVECs were transfected with si-circ3302, and then treated with medium containing 15% sera from HC (*n* = 6) and KD subjects (*n* = 6) for 48 h. Next, the expression of circ3302, Vimentin and ZO-1 was measured by RT-qPCR analysis. **d** Endothelial cells were treated as above, and then the protein levels of ZO-1, Claudin-1, Twist, Vimentin, and β-catenin were measured by western blot analysis. **e**, **f** Effect of circ3302 silencing on KD serum-induced migration was evaluated using scratch and transwell assays. Magnification:×40 and scale bar = 100 μm for scratch assay. Magnification:×100 and scale bar = 50 μm for transwell assay. *P < 0.05 vs. the HC group or KD group.

Above findings have demonstrated that upregulation of circ3302 promoted EndMT. To investigate whether downregulation of circ3302 has a therapeutic effect on KD-related EndMT, the expression of circ3302 was silenced in endothelial cells before treatment with KD sera (Fig. 4a). Results displayed that the mRNA level of Vimentin was decreased while ZO-1 was augmented compared with the KD group (Fig. 4b, c). In addition, silencing of circ3302 remarkably increased the expression of endothelial markers including ZO-1 and Claudin-1, and decreased the expression of mesenchymal-specific proteins such as Twist, Vimentin, and β-catenin at protein levels compared with KD group (Fig. 4d). Scratch and transwell assays revealed that circ3302 silencing could weaken KD sera-mediated migration ability (Fig. 4e, f). These data indicated that silencing of circ3302 alleviated KD-related EndMT.

### Circ3302 could modulate KIT expression

**Fig. 5:**
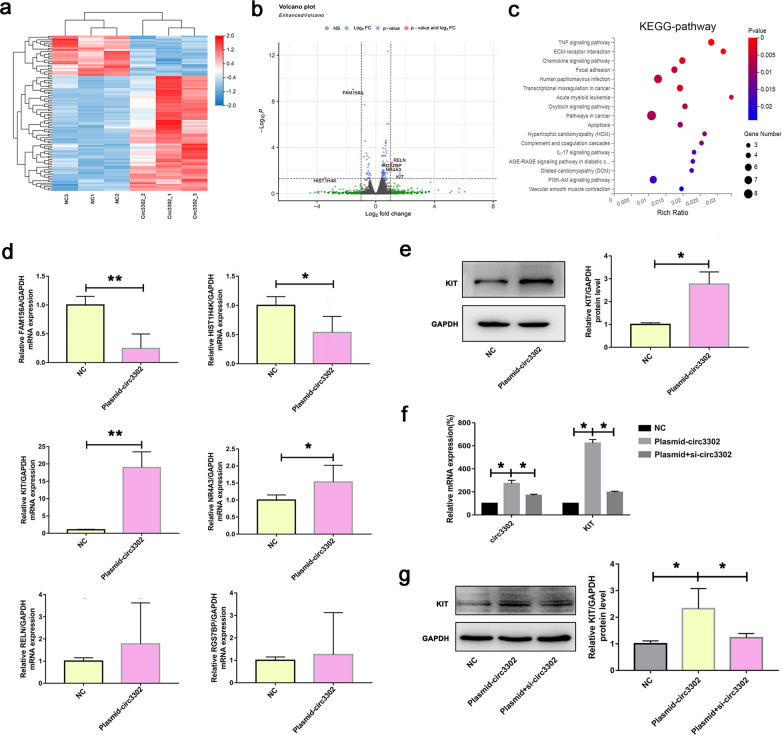
Changes of gene expression after circ3302 overexpression in HUVECs and the relationship between circ3302 and KIT. **a** Heatmap illustrated the gene expression profiling of HUVECs after circ3302 overexpression. **b** Gene expression profiling (Volcano plot) of HUVECs after circ3302 was overexpressed. **c** KEGG pathway analysis was conducted in HUVECs with circ3302 overexpression. **d** HUVECs were transfected with plasmid-circ3302, and the mRNAs levels of *FAM156A*, *HIST1H4K*, *KIT*, *NR4A3*, *RELN*, and *RGS7BP* were detected by RT-qPCR analysis. **e–g** After transfected with plasmid-circ3302 or plasmid-circ3302 plus si-circ3302, the expression of KIT was determined at mRNA levels and protein levels. Significance: *P < 0.05, and **P < 0.01 vs. the NC group or Plasmid-circ3302 group. Plasmid+si-circ3302, indicated plasmid-circ3302 plus si-circ3302.

To better distinguish the profile alterations of circ3302 modulating KD-associated EndMT, the RNA-seq analysis of endothelial cells that overexpressed circ3302 was conducted. As manifested in the heatmap (Fig. 5a), we found 92 differentially expressed genes, with 74% upregulated and 26% downregulated, among which 6 top differentially expressed genes were selected: *RGS7BP*, *RELN, NR4A3*, *KIT*, *FAM156A*, and *HIST1H4K* (Fig. 5b). KEGG pathway enrichment analysis displayed that circ3302 was mainly involved in TNF signaling pathway and ECM-receptor interactions (Fig. 5c), which are closely related to EndMT process [13, 29]. To further screen out the most likely target genes, we first determined the mRNA expression levels of *FAM156A*, *HISTIH4K*, *KIT*, *NR4A3*, *RGS7BP*, and *RELN* after circ3302 overexpression. The result showed that circ3302 overexpression downregulated the expression of *FAM156A* and *HISTIH4K*, and upregulated *KIT* and *NR4A3*. However, no significant change was found in the expression of RELN and RGS7BP (Fig. 5d). Previous studies reported that KIT upregulation can promote EMT in multiple cancers [30]. Therefore, we speculated that circ3302 induced EndMT via KIT. To confirm our speculation, we first verified KIT protein expression. Consistent with the mRNA levels, circ3302 overexpression significantly increased KIT expression at the protein level (Fig. 5e). To further substantiate that circ3302 could modulate KIT expression, we silenced circ3302 after its overexpression in endothelial cells. As expected, silencing of circ3302 remarkably decreased KIT expression at both mRNA and protein levels compared with circ3302-overexpressing endothelial cells (Fig. 5f, g). Taken together, these data indicated that circ3302 could regulate KIT expression.

### Inhibiting the expression of KIT suppressed KD serum-induced EndMT

**Fig. 6:**
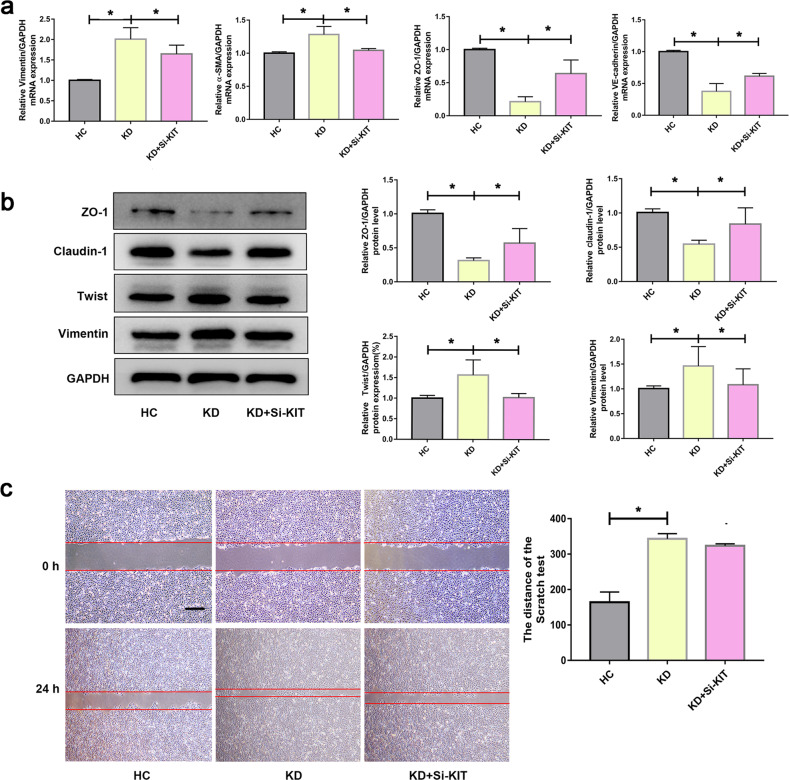
Knowing down of KIT alleviates KD serum-mediated EndMT process. **a** HUVECs were transfected with si-KIT, and then treated with medium containing 15% sera from HC (*n* = 6) and KD subjects (*n* = 6) for 48 h. After that, the mRNA levels of Vimentin, α-SMA, ZO-1, and VE-cadherin were examined by RT-qPCR analysis. **b** The protein levels of ZO-1, Claudin-1, Twist, and Vimentin were determined by Western blotting. **c** Migration ability was analyzed by scratch assay (n = 3) Magnification:×40 and scale bar = 100 μm for scratch assay. Data were shown as mean ± SD, and *P < 0.05 vs. the HC group or KD group.

The above data have demonstrated that circ3302 could modulate KD-related EndMT, and KIT could regulate circ3302-mediated EndMT (Fig. S3). To further investigate whether KIT could affect KD-associated EndMT, KIT expression was knocked down. Observation showed that KIT silencing significantly decreased the expression of Vimentin and α-SMA, and increased ZO-1 and VE-cadherin expression at mRNA levels compared with KD group (Fig. 6a). Moreover, we also observed the similar changes at the protein levels (Fig. 6b). However, KIT knockout had no significant effect on migration induced by KD serum (Fig. 6c). These results suggest that silencing KIT partially alleviated KD-associated EndMT.

### Circ3302 regulated KIT expression via binding miR-135b-5p in KD

**Fig. 7:**
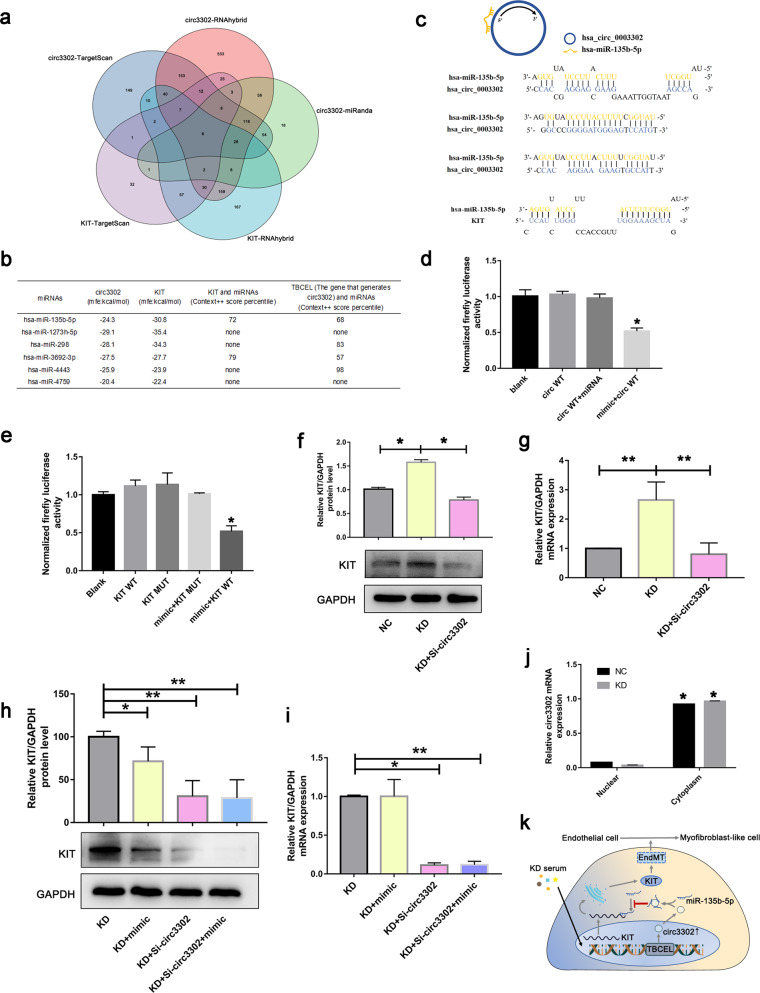
Circ3302 bound miR-135b-5p to regulate KIT expression. **a** TargetScan, miRanda and RNAhybrid were used to predict the potential miRNAs that bind to circ3302. TargetScan and RNAhybrid were utilized to screen potential miRNAs that target KIT. Then miRNAs that both bind to circ3302 and target KIT were further obtained via Venn diagram. **b** BiBiServ2 and TargetScan were used to predict the binding capacity and scores of circRNAs/KIT and miRNAs. **c** The predicted binding sites between circ3302 and miR-135b-5p, and between miR-135b-5p and KIT. **d**, **e** Luciferase results between circ3302 and miR-135b-5p, and between KIT and miR-135b-5p. Data were presented as mean ± SD (*n* = 3), and *P < 0.05 vs. the blank group. **f**, **g** The protein and mRNA levels of KIT were respectively examined in HC group, KD group, and KD + Si-circ3302 group. Data were shown as mean ± SD (*n* = 3), and *P < 0.05 and **P < 0.01 vs. the HC group or KD group. **h**, **i** The protein and mRNA expression of KIT was examined in KD group, KD + mimic group, KD + Si-circ3302 group, and KD + Si-circ3302 + mimic group. **j** Distribution of circ3302 was analyzed in the nucleus and cytoplasm. Data were expressed as mean ± SD (*n* = 3), and *P < 0.05 vs. the HC Nuclear group or KD Nuclear group. **k** Schematic diagram about the mechanism of circ3302 inducing EndMT process. KD serum increased the expression of circ3302, which then bound to miR-135b-5p to inhibit its binding with KIT mRNA, finally resulting in elevated KIT translation and subsequent EndMT in KD.

To further explore the mechanism of circ3302 regulating KIT, we used three independent sources of miRNA target predictions (TargetScan, miRanda and RNAhybrid) to predict potential miRNAs that bind to circ3302. TargetScan and RNAhybrid were utilized to screen miRNAs that target KIT. Next, miRNAs that both bind to circ3302 and target KIT were further obtained. As shown in Fig. 7a, the Venn diagram displayed 6 miRNAs that both bind to circ3302 and target KIT. Then we predicted the binding power (mfe) and the context score percentile of these 6 miRNAs on the website (Fig. 7b). We got 2 most likely miRNAs from the table including miR-135b-5p and miR-3692-3p. Next, we verified the predicted results via the luciferase experiment. Data showed that miR-135b-5p might be the potential miRNA (Fig. S4). Further analysis found that there were binding sites between circ3302 and miR-135b-5p (Fig. 7c). Moreover, luciferase assays also demonstrated that the mutual interactions between circ3302 and miR-135b-5p (Fig. 7d). The binding between KIT and miR-135b-5p were postulated via RNAhybrid website (Fig. 7c) and confirmed by luciferase assay (Fig. 7e). To better illustrate the mechanism by which circ3302 binds miR-135b-5p to regulate KIT expression in KD condition, we measured the expression levels of KIT. Results showed that KIT expression was significantly upregulated after treatment with KD serum, while si-circ3302 could reverse its upregulation (Fig. 7f). Moreover, we observed the similar result at the mRNA levels (Fig. 7g). Further study showed that KIT protein expression was significantly decreased by upregulation of miR-135b-5p, and further reduced after simultaneously down-regulating circ3302 compared with the KD group (Fig. 7h). However, the mRNA level of KIT was not affected by the upregulation of miR-135b-5p compared with KD group (Fig. 7i), indicating that miR-135b-5p interrupted KIT translation process. To explore the mechanism, the localization of cir3302 was determined. As shown in Fig. 7j, circ3302 was expressed in both nucleus and cytoplasm, but mainly enriched in the cytoplasm. Taken together, these results indicated that circ3302 might bind to miR-135b-5p to weaken its inhibitory effect on KIT, promoting KIT translation and KIT-induced EndMT (Fig. 7k).

## Discussion

The EndMT process regulates the damage of the vascular endothelium in the acute phase of KD and the formation of coronary artery aneurysms in the later stage [6]. Therefore, exploring the actual molecular mechanism of EndMT provides new insights into the clinical medication of KD.

Accumulating studies have demonstrated that non-coding RNAs (including lncRNAs, miRNAs) play an indispensable role in the EndMT. For example, Monteiro et al. demonstrated that loss of MIR503HG promotes EndMT transition in vascular disease [31]. He et al. reported that miR-483 targeting of CTGF suppresses EndMT in KD [6]. In addition, studies have illustrated that circRNAs significantly regulate diverse biological and pathological processes [32–34]. However, the significant effect of circRNAs on KD-associated EndMT remains elusive. In this study, we for the first time demonstrated that the expression of circRNAs was differential in endothelial cells treated by KD serum compared with HC serum, screened out circ3302 and explored its molecular mechanism of regulating EndMT in KD.

In our study, we substantiated the upregulation of circ3302 in KD serum-treated endothelial cells, and demonstrated that circ3302 could induce EndMT as evidenced by the decreased expression of endothelial markers and increased expression of EndMT-related indicators. However, overexpression of circ3302 had no effect on migration ability, which was not consistent with KD serum-treated endothelial cells, indicating that circ3302 may partially contribute to KD serum-induced EndMT. Subsequent studies showed that silencing of circ3302 significantly alleviated KD serum-induced EndMT as demonstrated by decreased expression of mesenchymal markers and increased level of endothelial markers, and decreased migration ability compared with KD group. These data revealed that KD serum-induced EndMT cannot be fully attributed to upregulation of circ3302, but silencing circ3302 could indeed mitigate KD-induced EndMT process. This could be explained by that circ3302 is a necessary but insufficient element to fully replicate KD-associated EndMT.

To further explore the molecular mechanism, RNA-seq analysis was conducted in endothelial cells that overexpressed circ3302. Six genes, including *RGS7BP*, *RELN, NR4A3, KIT*, *FAM156A*, and *HIST1H4K*, were screened out. The RT-qPCR analysis confirmed that *FAM156A* and *HIST1H4K* were significantly downregulated, and *KIT* and *NR4A3* were notably upregulated in circ3302-overexpressing endothelial cells. FAM156A, also named TMEM29, is demonstrated to be related to apoptotic cell death especially at the time points of caspase 3 activation [35]. Methylation of HIST1H4K exhibits a significant correlation with aging [36]. A pioneering research indicates that NR4A3 inhibition significantly promotes epithelial-mesenchymal transition (EMT), proliferation, migration and invasion in tumor cells [37]. Emerging evidence suggests that KIT is a tyrosine kinase receptor mainly involved regulating hematopoiesis, lung, heart, nervous system, which further regulates proliferation, differentiation, migration, and migration cell survival [38, 39]. Moreover, activation of KIT is able to promote EMT phenomenon in cancers [30]. We assumed that circ3302 might induce EndMT via activating KIT. Our following experiments also substantiated that circ3302 regulated EndMT process through KIT (Figure S3). Moreover, silencing of KIT could mitigate KD serum-induced EndMT process, similar to circ3302 silencing, manifesting that downregulation of circ3302 alleviated KD-associated EndMT through modulating KIT.

Mounting evidence indicates that circRNAs play regulatory roles through acting as microRNA sponges or interacting with proteins to regulate selective splicing or transcription, and epigenetic modification [16, 40, 41]. For instance, circHECW2 regulates the EndMT by directly binding to MIR30D, subsequently resulting in the activation of nonautophagic role of ATG5 [42]. To deeply investigate the mechanism of circ3302 regulating KIT, we combined sequencing results with prediction software to screen out miR-135b-5p. The follow-up experiments also verified that miR-135b-5p could interact with both circ3302 and KIT. Moreover, the KD serum-mediated KIT protein expression was significantly decreased by circ3302 silencing and miR-135b-5p. However, the KIT mRNA level was just regulated by circ3302, and not affected by miR-135b-5p. This result revealed that miR-135b-5p mainly affected KIT translation, in line with previous reports that microRNAs can repress mRNA translation rather than influence mRNA stability when incompletely complementary to the target genes [43]. In addition, these data also implicated that circ3302-mediated KIT regulation was not just via miR-135b-5p, and other mechanisms might be involved, which need further investigation. Our further study showed that circRNA was mainly expressed in the cytoplasm. All these results suggest that circ3302 induces EndMT by binding to miR-135b-5p in the cytoplasm and reducing the inhibitory effect on KIT translation to promote KIT expression. This is consistent with the report that circRNAs mainly bind miRNA in the cytoplasm to regulate post-transcriptional target proteins [44–46].

In conclusion, we first proved that circ3302 promoted KD-associated EndMT progression by inducing KIT expression by binding to miR-135b-5p. However, some limitations exist in our studies. First of all, whether circ3302 regulates the progression of EndMT via other biological processes, such as binding RNA-binding proteins (RBPs) or being translated into a peptide [47–52], which require further investigation. Secondly, we found that overexpression of circ3302 could not remarkably induce migration. However, downregulation of circ3302 in the KD group could alleviate migration. These data implied that KD serum-induced endothelial cell migration might result from multi-factors, not just circ3302, which needs further exploration. Thirdly, in this study, we just confirmed the upregulation of circ3302 in endothelial cells. Whether its expression is also changed in other cells such as smooth muscle cells or fibroblasts is still unclear. Nevertheless, our study substantiates that KD-related EndMT is associated with circ3302 upregulation and delivers an excellent therapeutic strategy.

## Materials and methods

### Research objects, specimen collection, and ethical considerations

The subjects participating in this study were KD patients and age-matched healthy controls who attended the Second Affiliated Hospital and Yuying Children’s Hospital of Wenzhou Medical University, Zhejiang province, China, from October 2019 to August 2021. The age range of KD patients and healthy controls was around 2–5 years old. Healthy controls (HCs) were children who have undergone regular health checkups and were free from infection. All KD patients met the standards set by the American Heart Association in 2017 and had coronary aneurysms but no other serious complications or underlying diseases. In addition, all KD patients planned to receive intravenous immunoglobulin (IVIG, 2 g/kg) plus aspirin (30-50 mg/kg/d) in our experiment. 500-μl serum samples from each KD patient were collected in the acute phase (before IVIG treatment on day 3 and day 7) and all serum samples were stored at −80 °C within 4 hours after collection until later use. All participants agreed in writing to use their clinical data and blood samples for academic research. This study was approved by the Ethics Committee of The Second Affiliated Hospital and Yuying Children’s Hospital of Wenzhou Medical University (LCKY2019-47) and was conducted according to the Declaration of Helsinki.

### Cell culture and treatment

Human umbilical vein endothelial cells (HUVECs) were purchased from Cybertron Biotechnology Co., Ltd (Shanghai, China). The cells were identified by short tandem repeat (STR) markers and no mycoplasma contamination was detected. HUVECs were routinely cultured in Endothelial Cell Medium (ECM) at 37 °C in a humidified incubator with a continuous 5% CO_2_ supply. HUVECs were incubated in different plates, including 6-well, 12-well, 24-well, and 96-well plates, for RT-qPCR analysis, Western blotting, scratch test, transwell migration assay, intracellular and extracellular RNA separation, luciferase experiment, etc. Serum-Free Cryopreservation Solution (C40100, NEW Cell & Molecular Biotech) was utilized for cell cryopreservation. HUVECs subjected to 2-10 passages were used in this study.

### Sequencing

HC or KD sera-treated cells were sent to Guangzhou Ruibo Company for circRNAs sequencing. The target circRNA was then analyzed and screened. In this study, we confirmed the different expression of circRNAs in HC or KD-treated HUVECs at a threshold of P-value <0.05 and fold-change > 2. Then, HUVECs were transfected with the upregulated circRNA and sent to BGI for transcriptome sequencing (repeated 3 samples).

### Western blot analysis

Heart tissues and cultured HUVECs were homogenized in RIPA buffer solution supplied with protease and phosphatase blockers. Then equal amounts of proteins (20-40 μg) were separated by 7.5% and 10% SDS-PAGE gels and electrotransferred onto the PVDF membrane (Bio-Rad). After blocking with 5% skim milk for 2 h, the membrane was incubated with the specific primary antibodies overnight at 4°C. The used primary antibodies were listed as follows: Vimentin (ab92547, Abcam, dilution: 1:1000), Claudin-1(ab211737, Abcam, dilution: 1:1000), β-catenin (#8480S, CST, dilution: 1:1000), VE-cadherin (#2158, CST, dilution: 1:1000), GAPDH (#5174, CST, dilution: 1:1000), ZO-1(#AF5145, Affinity, dilution: 1:1000), Snail(#AF6032, Affinity, dilution: 1:1000), Twist1(#AF4009, Affinity, dilution: 1:1000), KIT (347321, ZenBio, dilution: 1:800). Afterward, the membranes were treated with the appropriate HRP-labeled secondary antibody for 2 h at room temperature. The protein bands were finally visualized using an enhanced ECL commercial kit and measured using ImageJ software (Version 4.1).

### Real-time quantitative polymerase chain reaction (RT-qPCR)

Total RNA was extracted with TRIzol reagent (Invitrogen, Carlsbad, CA, USA), and the overall quality was assayed by agarose gel electrophoresis. The cDNA was obtained using a PrimeScript™ RT Reagent Kit with gDNA Eraser (Takara). Next, real-time PCR was conducted with Applied Biosystems QuantStudio 3 real-time PCR system (ThermoFisher) using Power TB green PCR Master Mix (Takara). Glyceraldehyde 3-phosphate dehydrogenase (GAPDH) and U6 were employed as internal controls. The 2^−ΔΔCT^ method was utilized to determine specific gene expression. Primers used in this study were shown in Table 1.

**Table 1:** List of primers used in this study.

Genes	Primers
GAPDH	Forward primer: CAAGGCTGTGGGCAAGGTCATC
	Reverse primer: GTGTCGCTGTTGAAGTCAGAGGAG
Vimentin	Forward primer: GCCCTAGACGAACTGGGTC
	Reverse primer: GGCTGCAACTGCCTAATGAG
ZO-1	Forward primer: CCTTCAGCTGTGGAAGAGGA
	Reverse primer: TGCTCAACTCCTTCGGGAAT
Hsa-circ3302	Forward primer: AGGAAGAAGTGCCATTCAGCAT
	Reverse primer: TGTGGCTGGGACATGGACTC
Hsa-circ7632	Forward primer: CATAGTACAGGCATCACAGAGAACC
	Reverse primer: CCAGCTGCATACAGAAGACTGTC
Hsa-circ4571	Forward primer: AATCATAAGAGCCATGGGACG
	Reverse primer: TTTACCAGATTCTCCAGCACTGC
Hsa-circ24609	Forward primer: CCCAGTAGTGACAATGCAGATGTT
	Reverse primer: GGGTCAAAATCATGTGATGCA
Hsa-circ2961	Forward primer: AAGGAGTTGGAGTGACCGTT
	Reverse primer: CGTGAACTGTTTTGCACTGCT
Hsa-circ50301	Forward primer: GGGTATTGCTGTCTCTAAGCCAGA
	Reverse primer: CCCTAATTGTCAGTGGTCCCTG
Hsa-circ80342	Forward primer: GAAAAGCCACAAGTTGACCAAGT
	Reverse primer: CTTTCCACTTTCTTTACGCTGCT
Hsa-circ3297	Forward primer: AAGGCAAATGGCAACAGGTCT
	Reverse primer: GATGAGTCGTAATTTCCAAGAAGG
KIT	Forward primer: ACCGTCTCCACCATCCATCCATC
	Reverse primer: ACCAGCGTGTCGTTGTCTTCTTTC
FAM56A	Forward primer: CCGAAGGCACCGCTGAATGG
	Reverse primer: GCTGTTCTGAGTACGAAGGCTGAG
HIST1H4K	Forward primer: GCAAAGGCGGGAAGGGTCTTG
	Reverse primer: GCTTGGCGTGCTCTGTATAGGTC
NR4A3	Forward primer: CGTCCGCTCCTCCTACACTCTC
	Reverse primer: TGGTGGTGGTGGTGGTGATGG
RELN	Forward primer: CAGGTCCCAAGCCACTCGTTTC
	Reverse primer: CTCCGTTCACAGTCAGCCAGTTC
RGS7BP	Forward primer: AACACCTGCCCTAGAAGACTCCTC
	Reverse primer: TGAGACAACACAGCCCAAAGAACC
α-SMA	Forward primer: CAGGGCTGTTTTCCCATCCAT
	Reverse primer: GCCATGTTCTATCGGGTACTTC
VE-Cadherin	Forward primer: CGGACGATGATGTGAACACC
	Reverse primer: TTGCTGTTGTGCTTAACCCC

### Construction of plasmid and si-RNA

Si-circ3302 and si-KIT were designed and synthesized by Guangzhou Ruibo Company, and plsamid circ3302 was constructed and synthesized by Shanghai Heyuan Biotechnology Company. Lipofecamine 3000 (Invitrogen, Carlsbad, CA, USA) and opti medium were used to transfect Si-circ3302, si-KIT and plsamid circ3302.

### Scratch test and transwell migration assays

In the scratch test, each well was scratched with the head of a sterile pipettor (200 μl) after the cell volume of the six-well plate reached more than 90%. Then the plate was washed with PBS and cultured with the serum- and sugar-free medium. Photographing was conducted for the cells at the indicated time under an inverted microscope. This experiment was repeated at least three times.

Transwell migration assay was performed in 24-well transwell plates with 8-μm pore-size chambers purchased from Corning Costar. After transfection, the cell concentration of each group was adjusted to 2.5 × 10^5^/100 μl. 200 μl of cell suspension was placed in the upper chamber, and 500 μl of 20% FBS culture medium was put in the lower chamber. After being cultured for 8–12 h (time varies with different groups), the cells were fixed with 4% paraformaldehyde, stained with crystal violet, washed with PBS.Then the cells that have not penetrated the membrane was wiped off with cotton swabs, and observed under a microscope.

### Nuclear and cytoplasmic separation

The total RNA from the nucleus and cytoplasm was extracted and isolated using the nuclear-cytoplasmic RNA-isolation kit. After reverse transcription, circ3302 expression was determined in the nucleus and the cytoplasm by RT-qPCR analysis.

### Luciferase reporter assay

The luciferase reporter plasmid using the pmirGLO dual-luciferase vector constructed by Guangzhou Ruibo Company. The corresponding plasmid and microRNA were co-transfected into HUVECs and the luciferase activity was evaluated using the dual-luciferase reporter kit (Promega, Madison, WI, USA). The relative activity of firefly luciferase was normalized to the activity of kidney luciferase. This experiment was used to reveal the interaction between circ3302, miR-135b-5p and KIT gene.

### Animal experiment

C57BL/6 mice (aged 3–4 weeks, average weight: 22–25 g) were purchased from Weitong Lihua Laboratory Animal Technology Co., Ltd. The mice were kept in separate cages in the animal room of the Animal Experiment Center of Wenzhou Medical University, with 22–24 °C temperature control, 50-60% humidity and a 12-h light–dark cycle. After a week of breeding, the KD model was constructed. The mice were randomly allocated into PBS and CAWS groups (*n* = 6 for each group), which were respectively injected with equal volumes of PBS or CAWS (4 mg/body) for 5 consecutive days, and continued regular feeding for 6 weeks. The mice were then sacrificed under anesthesia and their hearts were obtained for performing further experiments. All the experimental procedures and protocols were approved by the Animal Care and Use Committee of Wenzhou Medical University(wydw2017-0046).

### Hematoxylin and eosin (H&E) staining

Mice hearts were collected and fixed with 4% paraformaldehyde and embedded with paraffin. Five-µm thick heart sections were deparaffinized and stained with H&E solution after alcohol gradient hydration, and observed under light microscope.

### Immunofluorescence for in vitro experiments

The treated cells were harvested and fixed for 15 min in PBS containing 4% (w/v) paraformaldehyde, and then permeabilized with 0.3% Triton X-100. Next, cells were incubated in 10% BSA for 60 min, and incubated overnight with primary antibodies anti-ZO-1 (Affinity) or anti-Vimentin (Abcam) at 4 °C. The next day, cells were incubated with secondary antibodies conjugated at room temperature for 1 h followed by wash with PBS before being mounted using Fluorescence anti-quencher containing DAPI. Finally, the stained cells were observed under the light microscope.

### Immunofluorescence for in vivo experiments

The heart tissues were deparaffinize, antigen repaired after alcohol gradient hydration, then incubated in 10% BSA with 0.3% Triton X-100 for 60 min. Then the tissue slice was overnight incubated with primary antibodies anti-ZO-1 (Affinity) or anti-Vimentin (Abcam) at 4 °C. The next day, it was incubated with secondary antibodies conjugated at room temperature for 1 h followed by wash with PBS before being mounted using Fluorescence anti-quencher containing DAPI.

### Statistical analysis

IBM SPSS Statistics V21.0 software (USA) and GraphPad Prism 7.0 software were used for statistical analysis. Data were expressed as mean ± SD. Statistical difference between two groups was determined using an unpaired Student’s *t* test, and statistical difference among multiple groups were analyzed by the one-way analysis of variance (ANOVA) test. *P < 0.05 and **P < 0.01 were considered as statistically significant. The sample size was determined in accordance with previous literature [53, 54], which performed similar experiments to obtain significant results. The variance was similar between the groups that were statistically compared.

## Supplementary information

Supplementary Figures

Original Western blots

Agreement with the change of authorship

## Data Availability

CircRNA-Seq and RNA-Seq data were respectively available in BioProject # PRJNA790660 and PRJNA793389. Data supporting the present study are available from the corresponding author upon reasonable request.
