# Supplementary material for: CircRNA-3302 promotes endothelial-to-mesenchymal transition via sponging miR-135b-5p to enhance KIT expression in Kawasaki disease
Source: Cell Death Discov. 2022 Jun 29;8:299. doi: 10.1038/s41420-022-01092-4 (PMC9243129; doi:10.1038/s41420-022-01092-4)
Supplement: Supplementary file 1 — Supplementary Figures [file 41420_2022_1092_MOESM1_ESM.doc]

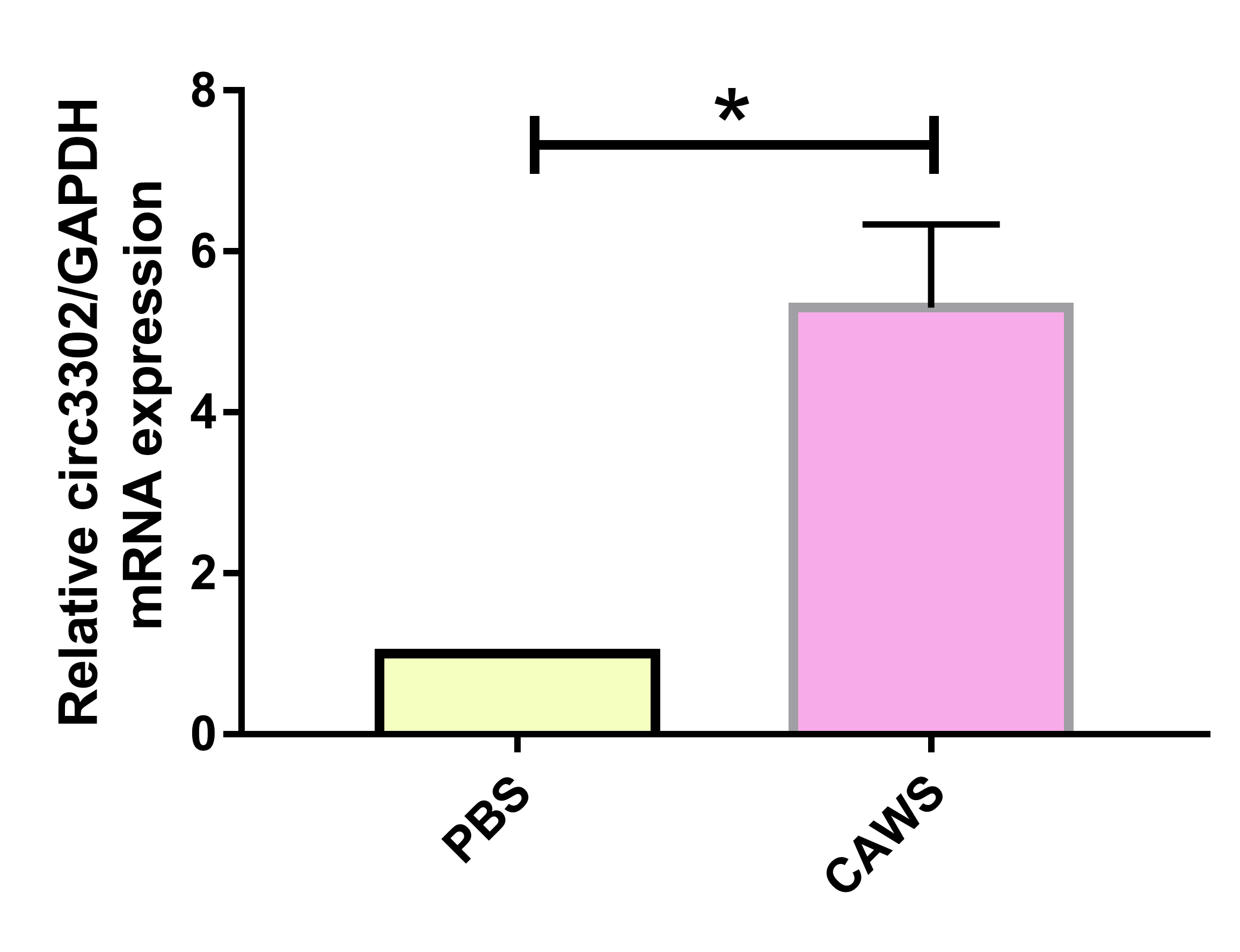


**Fig. S1 Circ3302 expression was determined in CAWS-induced KD mice model**. Data were shown as mean±SD (n=4). **P*<0.05 vs. PBS group.


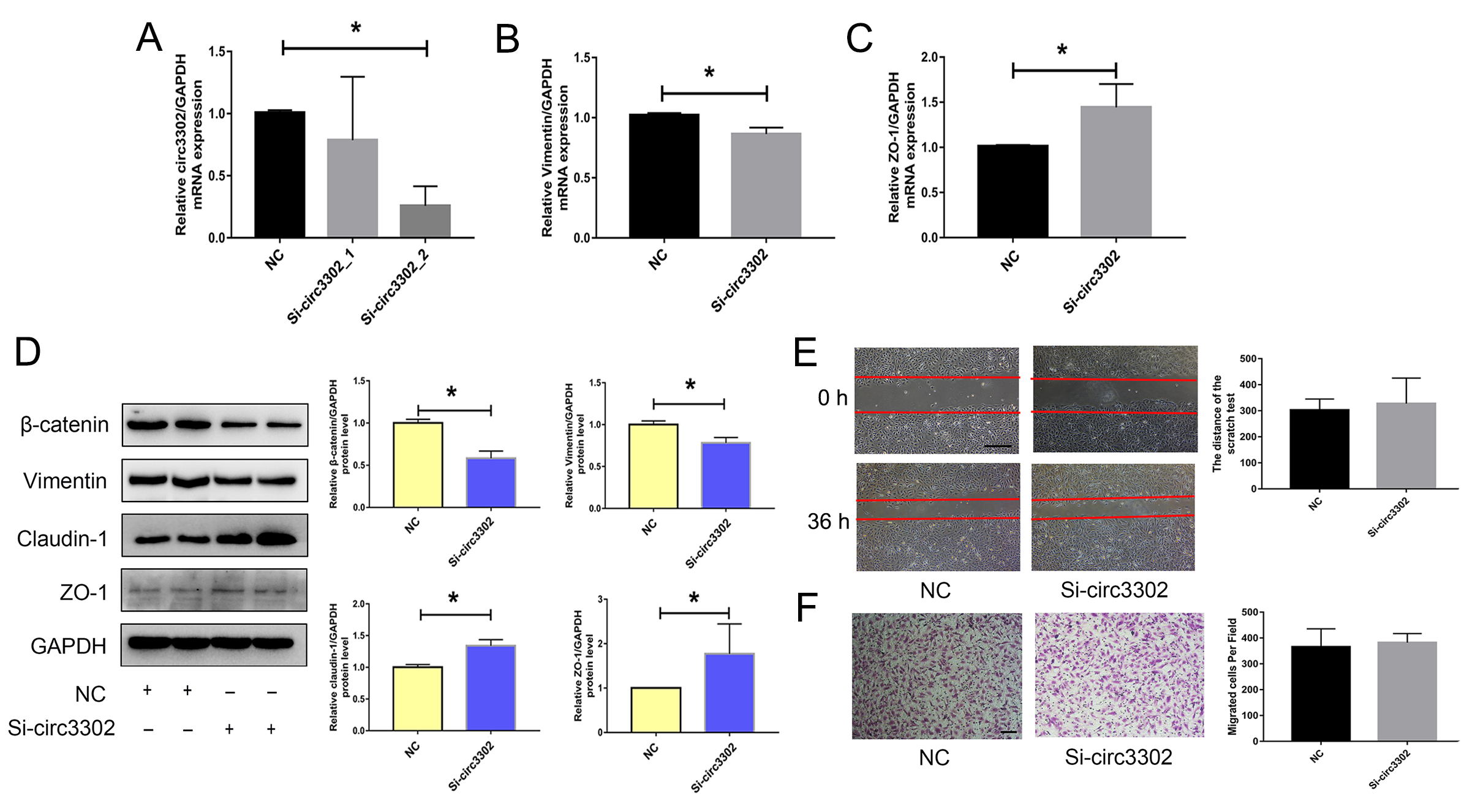


**Fig. S2 Circ3302 silencing increased the expression level of endothelial markers but decreased the expression of EndMT-related indicators.** (A)Screening si-circ3302 for the best down-regulation via RT-qPCR analysis. (B-C) HUVECs were transfected with si-circ3302, and then the mRNA level of Vimentin and ZO-1 were measured by RT-qPCR analysis. (D) HUVECs were transfected with si-circ3302, and the protein levels of β-catenin, Vimentin, Claudin-1 and ZO-1 were determined by Western blotting. (E-F) Effect of si-circ3302 on migration was respectively evaluated through scratch and transwell assays. Magnification:×40 and scale bar=100 μm for scratch assay. Magnification:×100 and scale bar=50 μm for transwell assay. Data were shown as mean±SD (n=3). **P*<0.05 vs. NC group.

**Fig. S3 Silencing of KIT alleviated circ3302-induced EndMT.** (A-B) Silencing of KIT significantly reversed Vimentin and ZO-1 expression mediated by Plasmid-circ3302 at mRNA levels. (C) KIT silencing remarkably affected Claudin-1 and Vimentin expression at protein level. Data were shown as mean±SD. **P*<0.05 vs. NC group or Plasmid-circ3302 group.


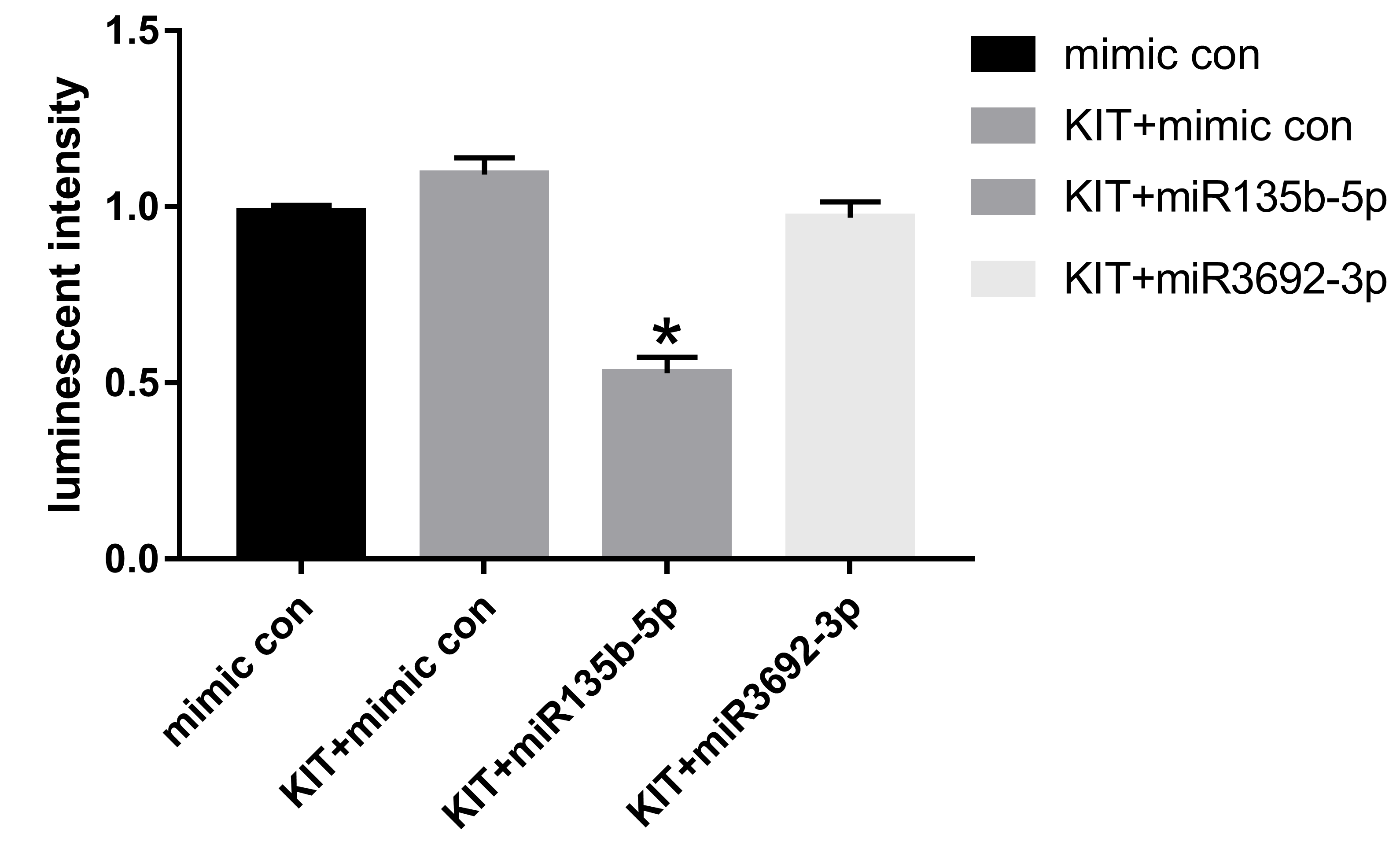


**Fig. S4 Screening of miRNAs via Luciferase reporter assay**. Data were exhibited as mean±SD (n=3). **P*<0.05 vs. Mimic con group.
