# Supplementary material for: CircRNA-3302 promotes endothelial-to-mesenchymal transition via sponging miR-135b-5p to enhance KIT expression in Kawasaki disease
Source: Cell Death Discov. 2022 Jun 29;8:299. doi: 10.1038/s41420-022-01092-4 (PMC9243129; doi:10.1038/s41420-022-01092-4)
Supplement: Supplementary file 2 — Original Western blots [file 41420_2022_1092_MOESM2_ESM.pdf]

Dear Editors,

Thank you very much for your information. Below are the original western blots. Due to these western blots were obtained within three years, we cropped the blots at that time to cut cost. But we can guarantee the shown blots are correct, and do not affect the conclusion. Owing the limited time, we can not re-carry out Western blotting in a short time. If we are lucky enough to have the chance to revise the manuscript, we will redo the Western blotting to obain the full and uncropped western blots. Wish the journal can give us the opportunity. Great thanks to you.

Best Regards

Yours Sincerely

Chang Jia

Fig.1a

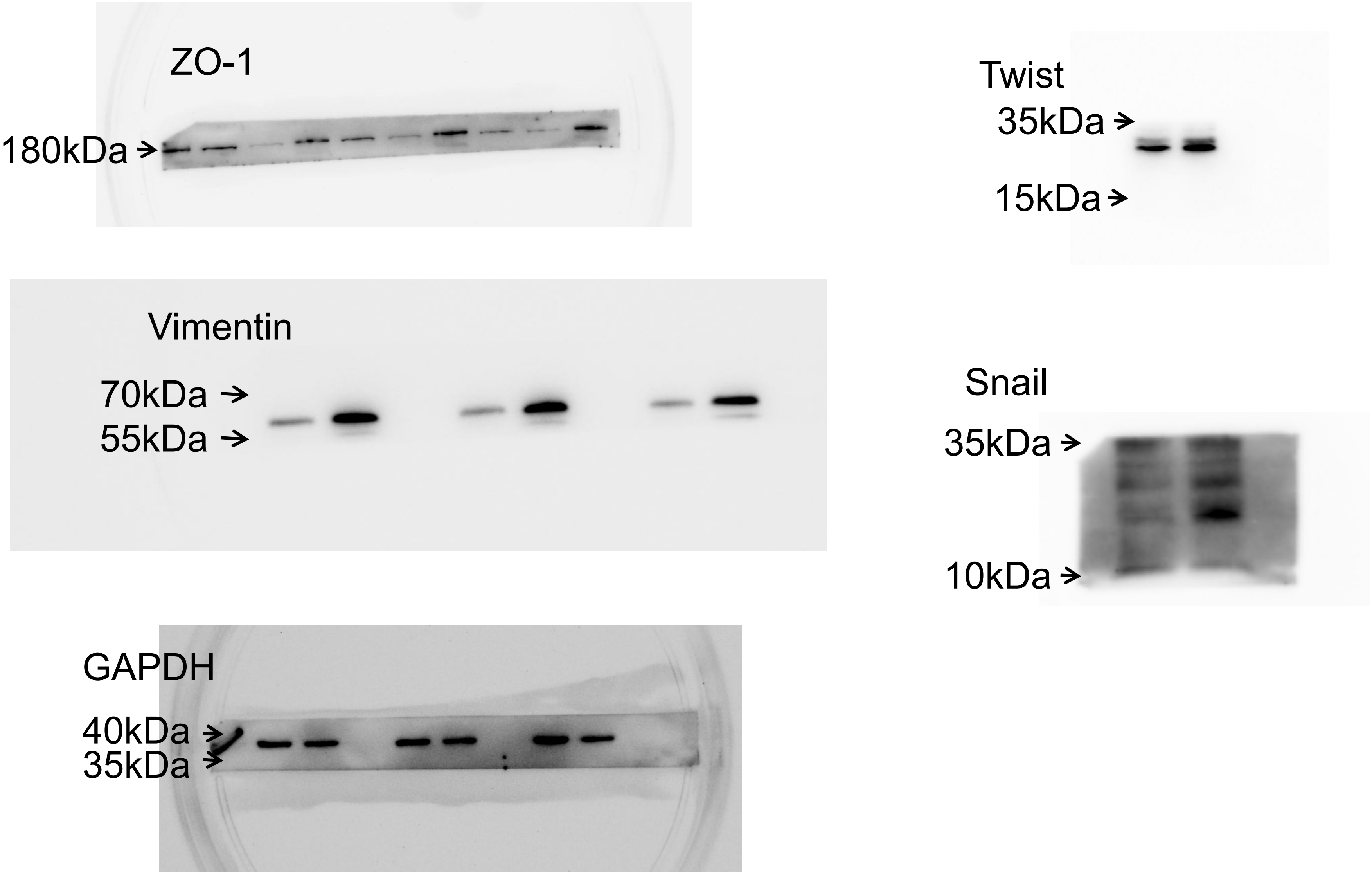
ZO-1
180kDa
Vimentin
70kDa
55kDa
GAPDH
40kDa
35kDa
Twist
35kDa
15kDa
Snail
35kDa
10kDa

Fig. 1g

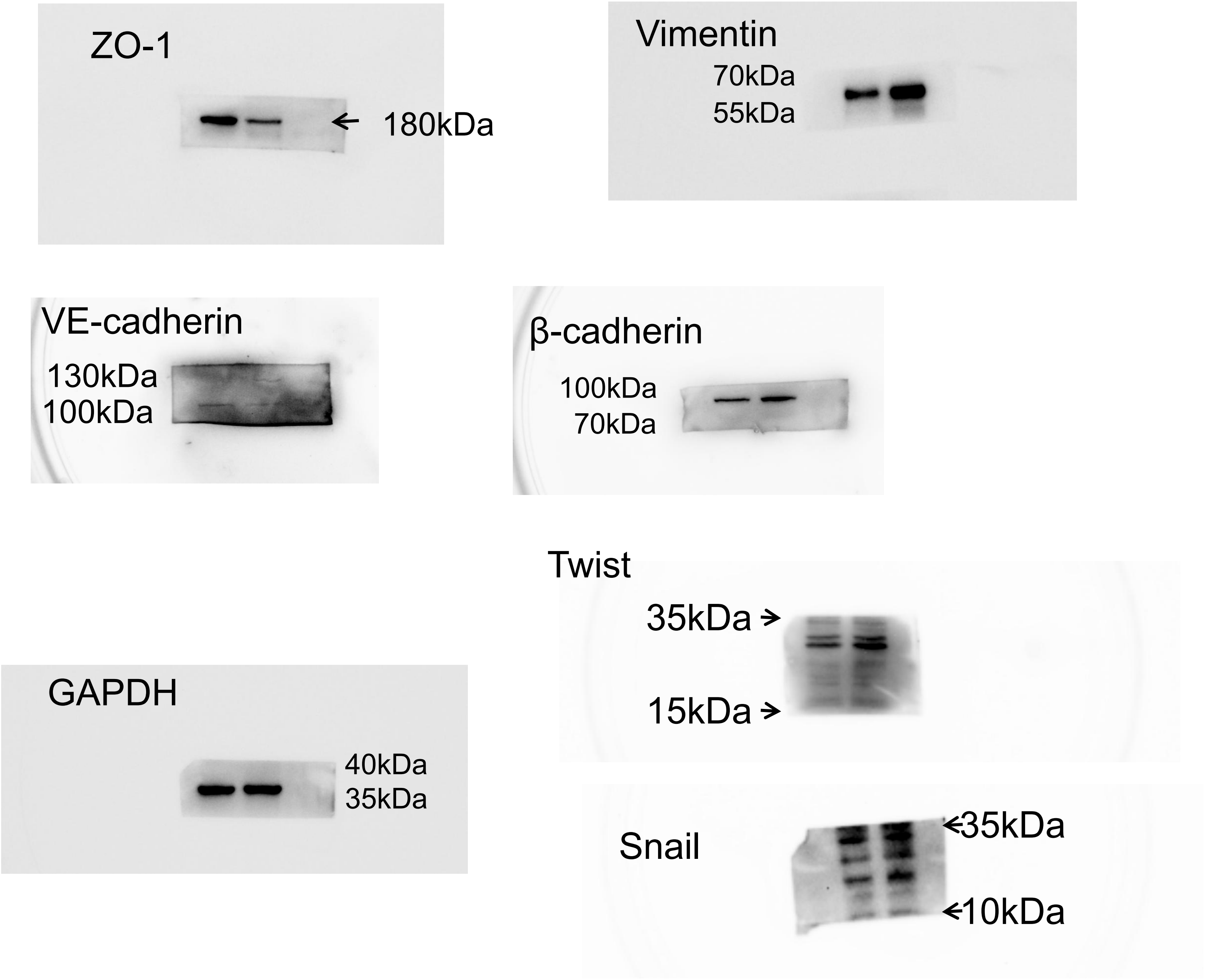
ZO-1
180kDa
Vimentin
70kDa
55kDa
VE-cadherin
130kDa
100kDa
β-cadherin
100kDa
70kDa
Twist
35kDa
15kDa
GAPDH
40kDa
35kDa
Snail
35kDa
10kDa

Fig. 3e

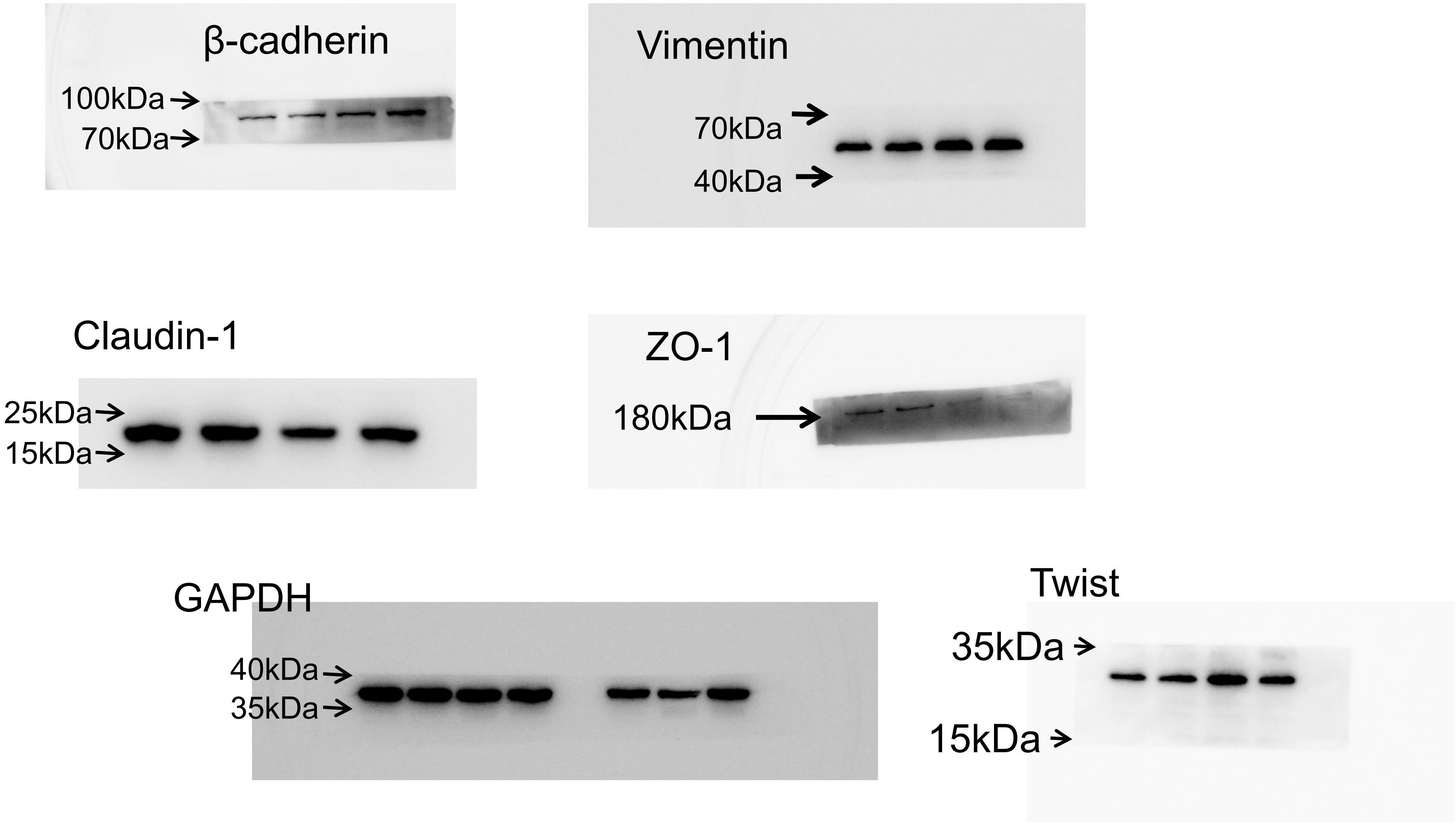

β-cadherin
100kDa
70kDa
Vimentin
70kDa
40kDa
Claudin-1
25kDa
15kDa
ZO-1
180kDa
GAPDH
40kDa
35kDa
Twist
35kDa
15kDa

Fig. 4d

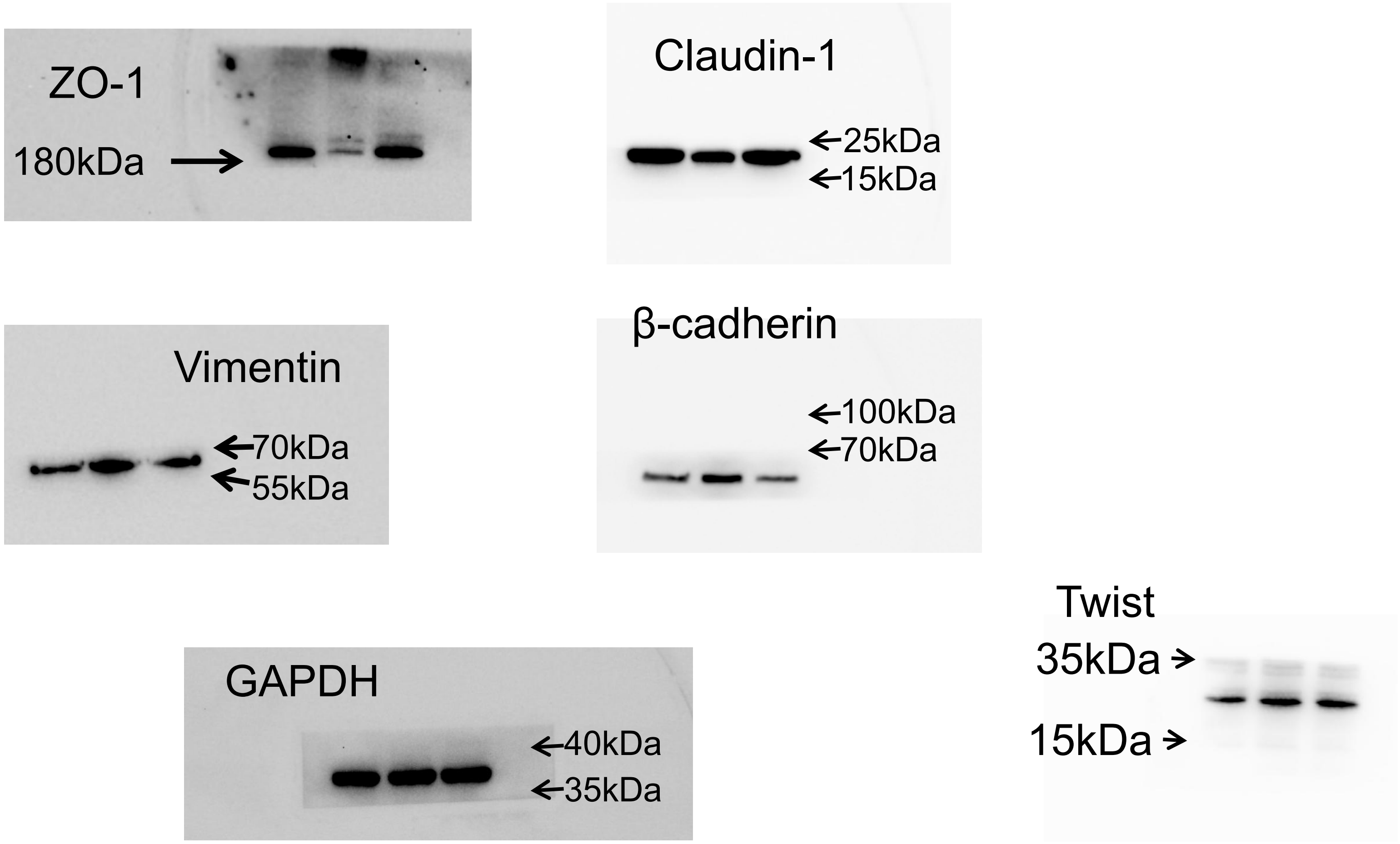
ZO-1
180kDa
Claudin-1
25kDa
15kDa
Vimentin
70kDa
55kDa
β-cadherin
100kDa
70kDa
Twist
35kDa
15kDa
GAPDH
40kDa
35kDa

Fig. 5e

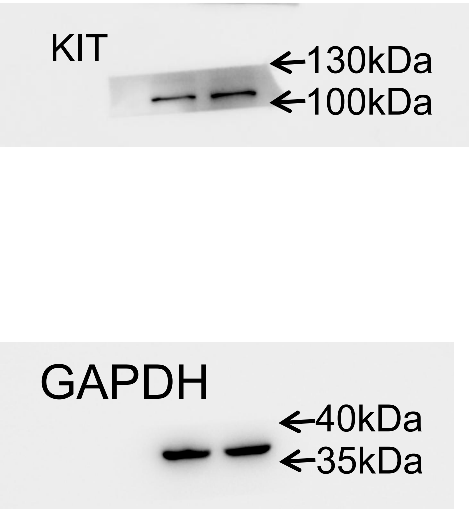

KIT
130kDa
100kDa
GAPDH
40kDa
35kDa

Fig.5 g

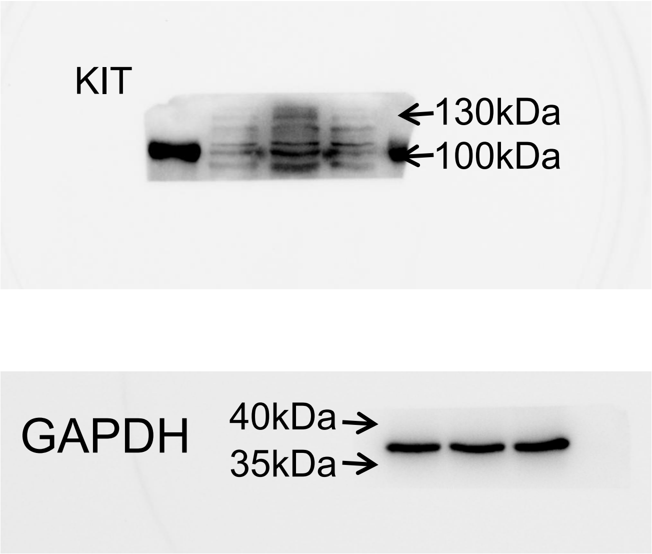

KIT
130kDa
100kDa
GAPDH
40kDa
35kDa

Fig.6b

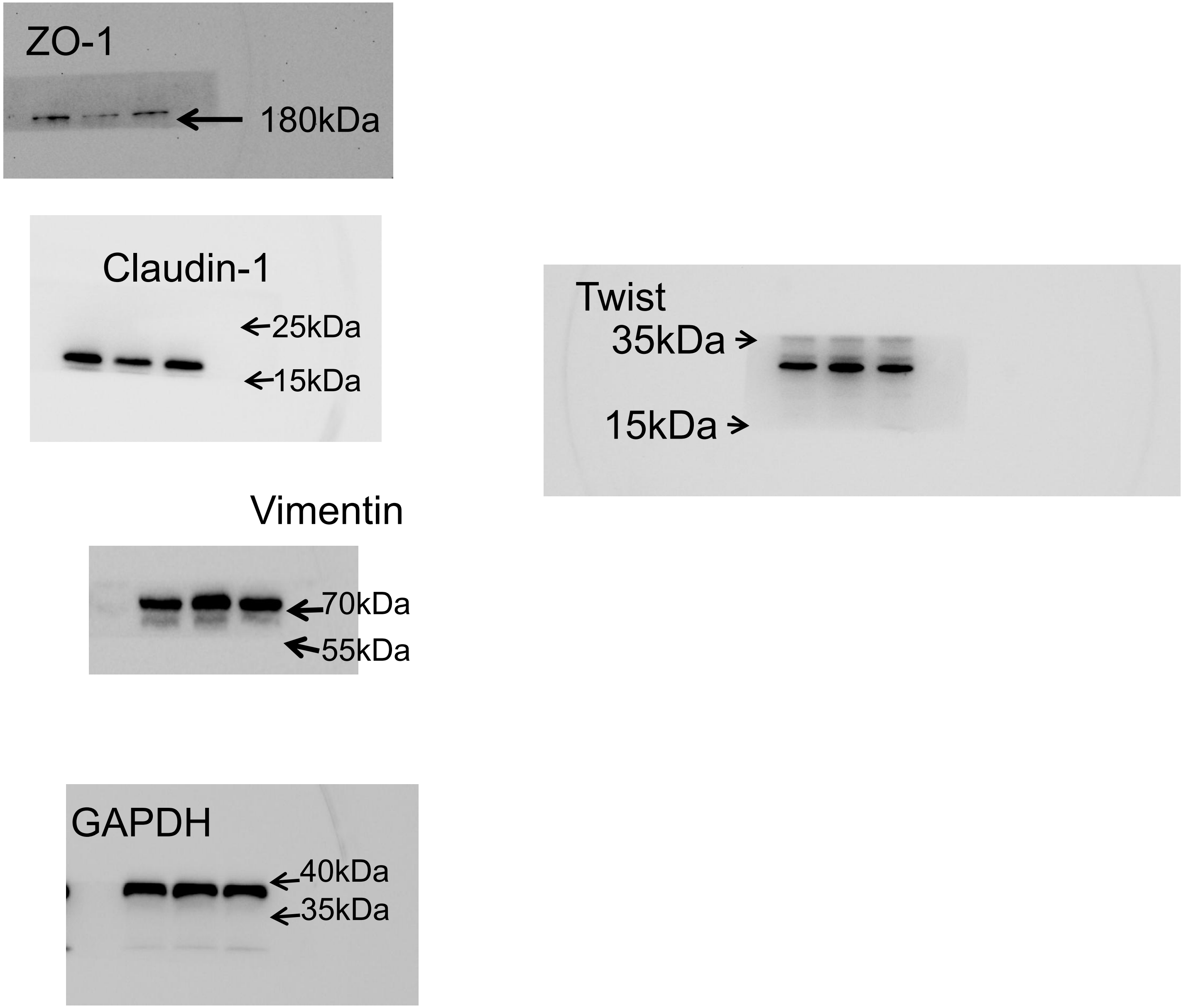
ZO-1
180kDa
Claudin-1
25kDa
15kDa
Vimentin
70kDa
55kDa
GAPDH
40kDa
35kDa
Twist
35kDa
15kDa

Fig.7f

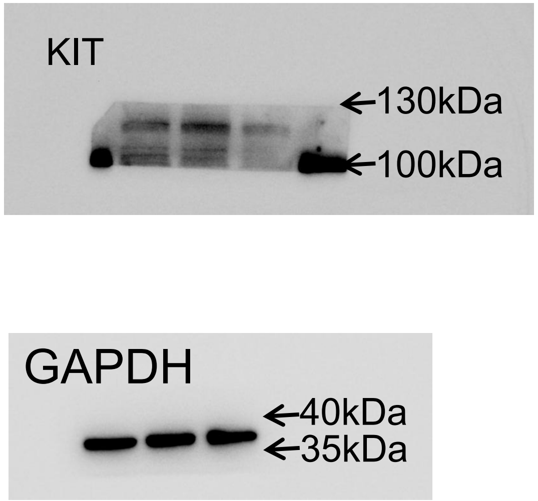
KIT
130kDa
100kDa
GAPDH
40kDa
35kDa

Fig.7h

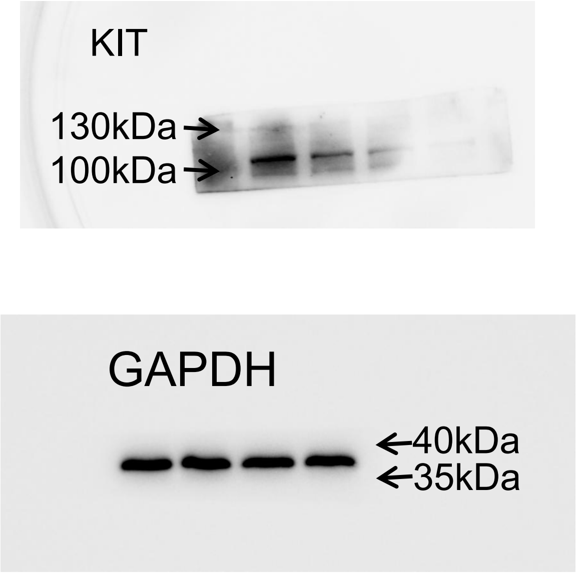
KIT
130kDa
100kDa
GAPDH
40kDa
35kDa

Fig. S2

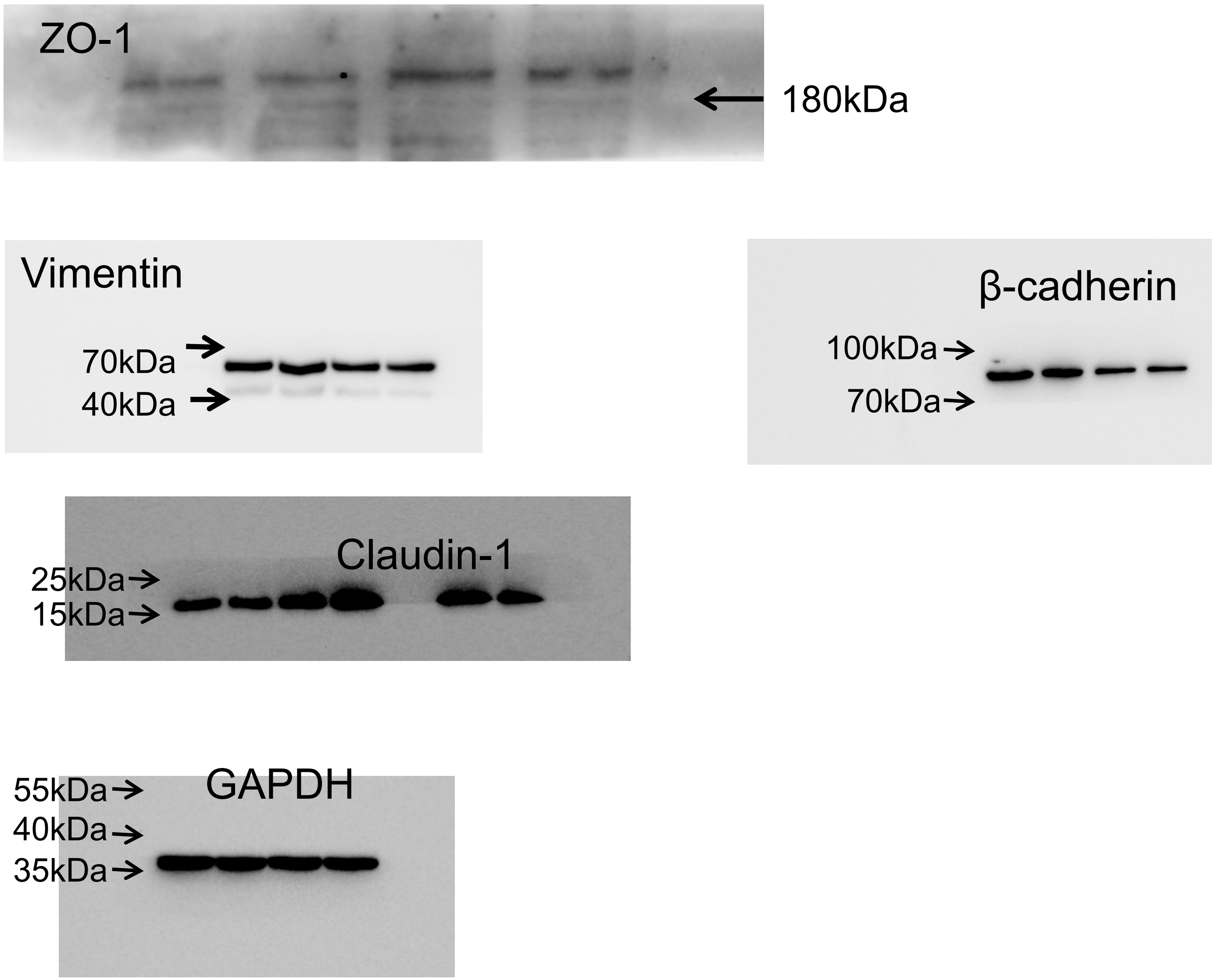
ZO-1
180kDa
Vimentin
70kDa
40kDa
β-cadherin
100kDa
70kDa
Claudin-1
25kDa
15kDa
GAPDH
55kDa
40kDa
35kDa

Fig. S3

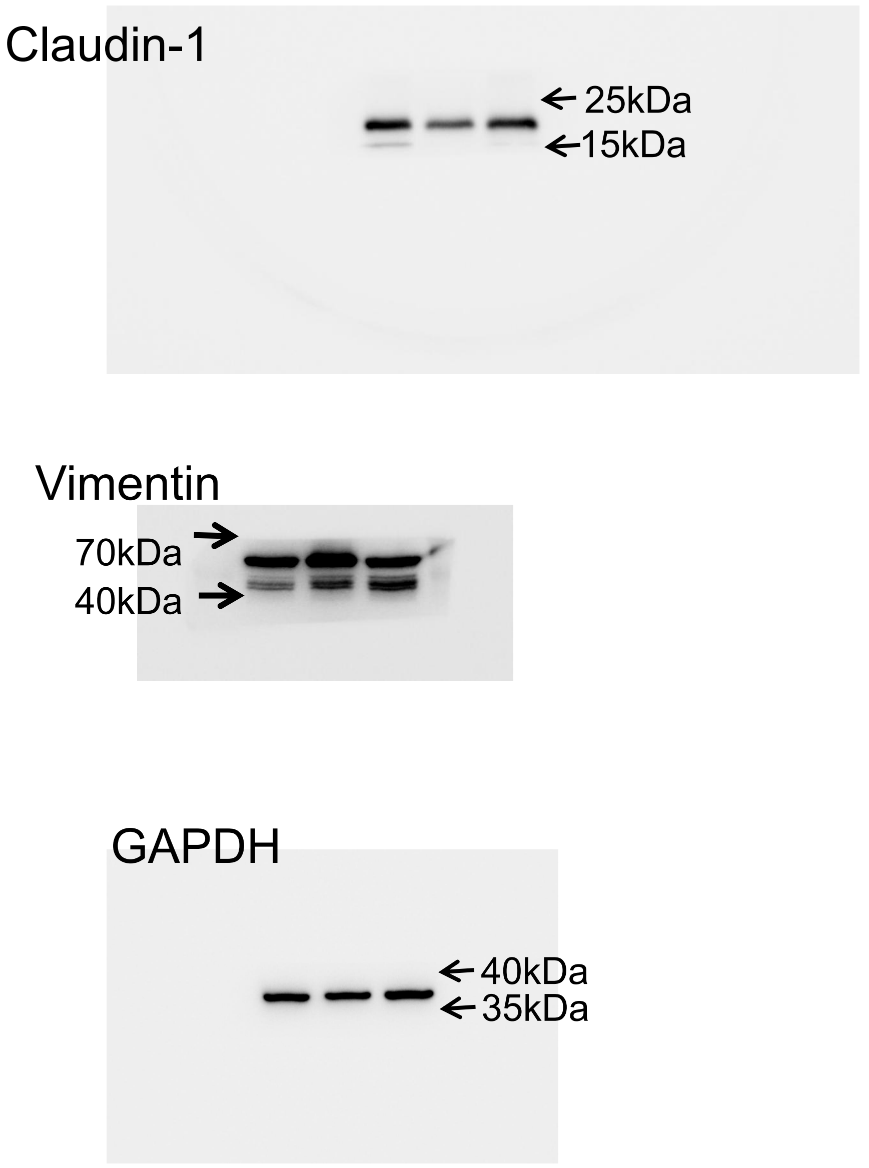

Claudin-1
25kDa
15kDa
Vimentin
70kDa
40kDa
GAPDH
40kDa
35kDa
