# Supplementary material for: CircRNA-3302 promotes endothelial-to-mesenchymal transition via sponging miR-135b-5p to enhance KIT expression in Kawasaki disease
Source: Cell Death Discov. 2022 Jun 29;8:299. doi: 10.1038/s41420-022-01092-4 (PMC9243129; doi:10.1038/s41420-022-01092-4)

**Chao Ni**


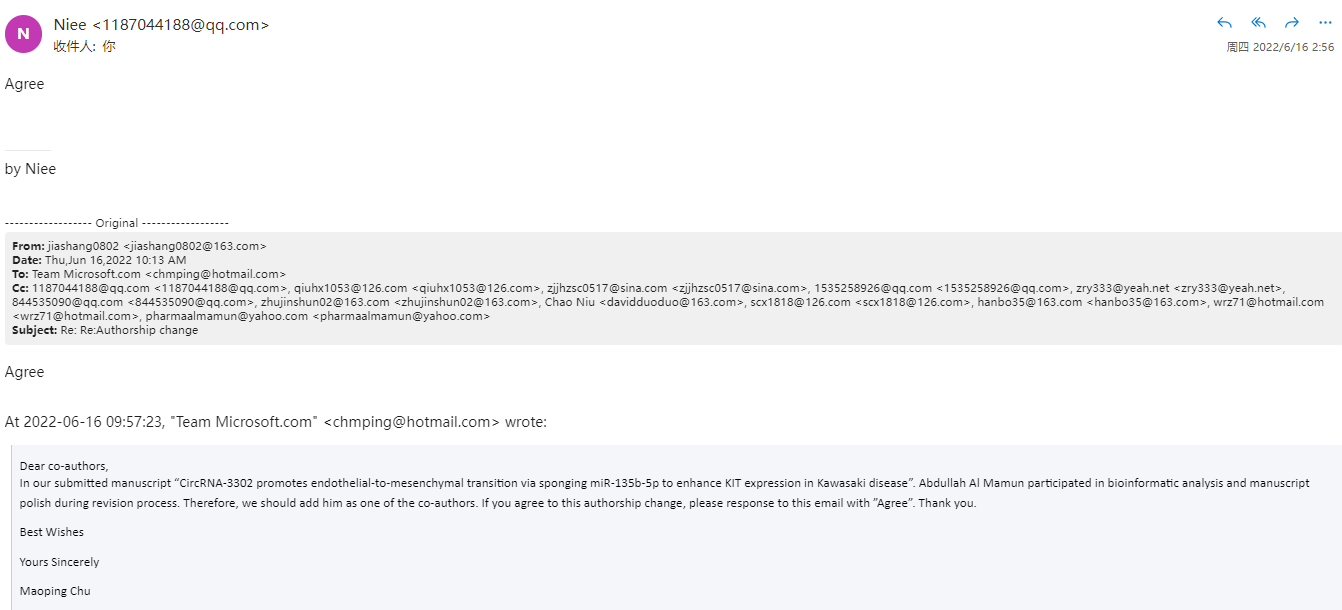


**Huixian Qiu**


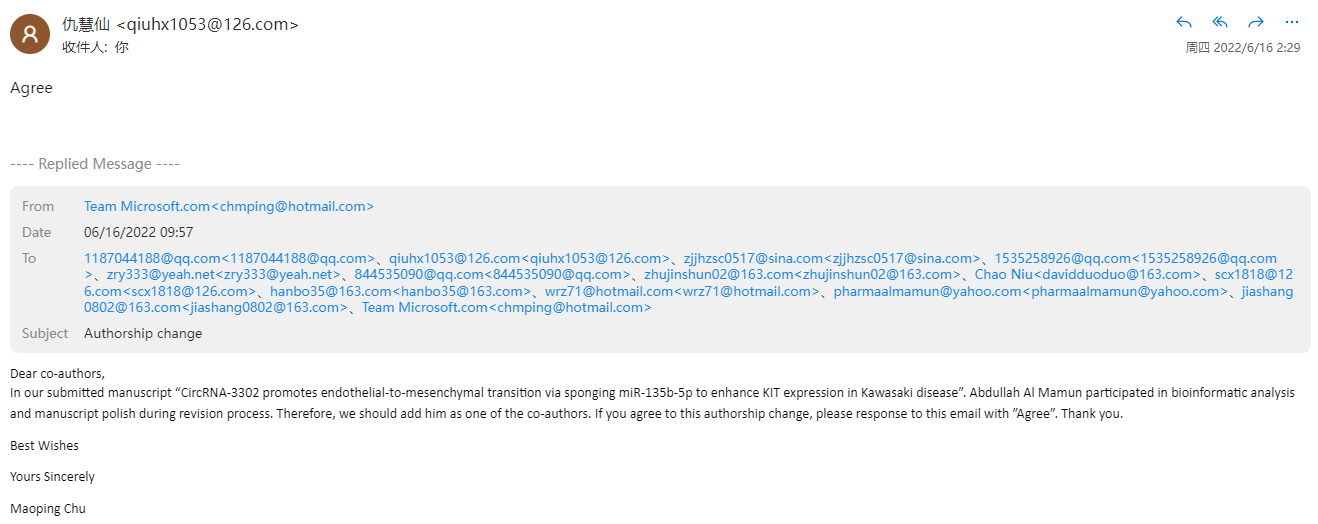


**Shuchi Zhang**


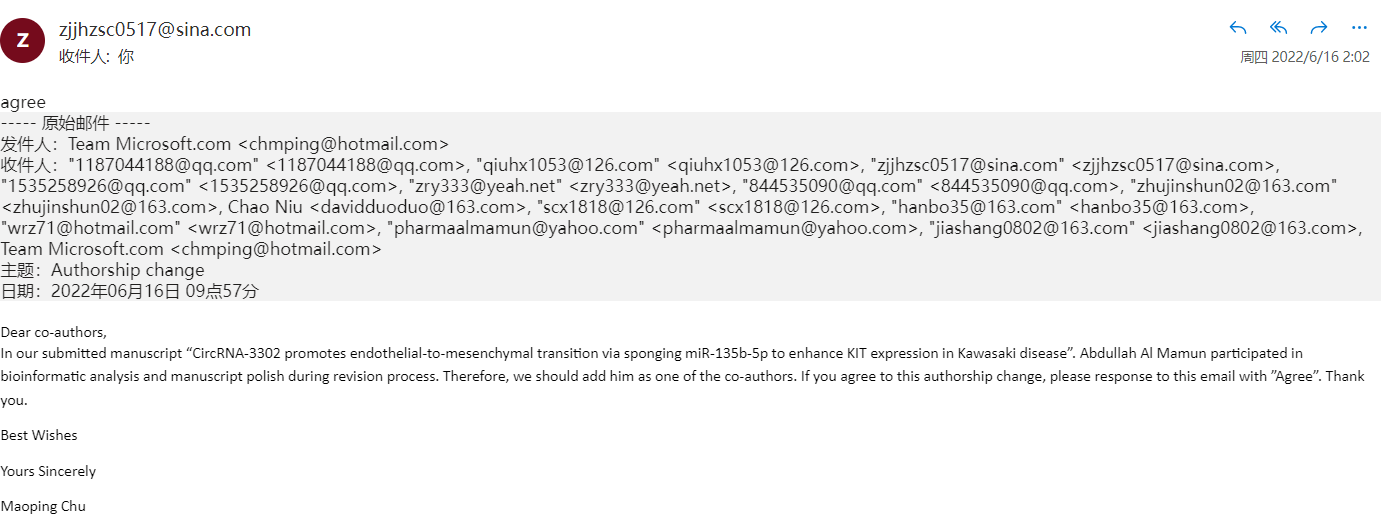


**Qihao Zhang**


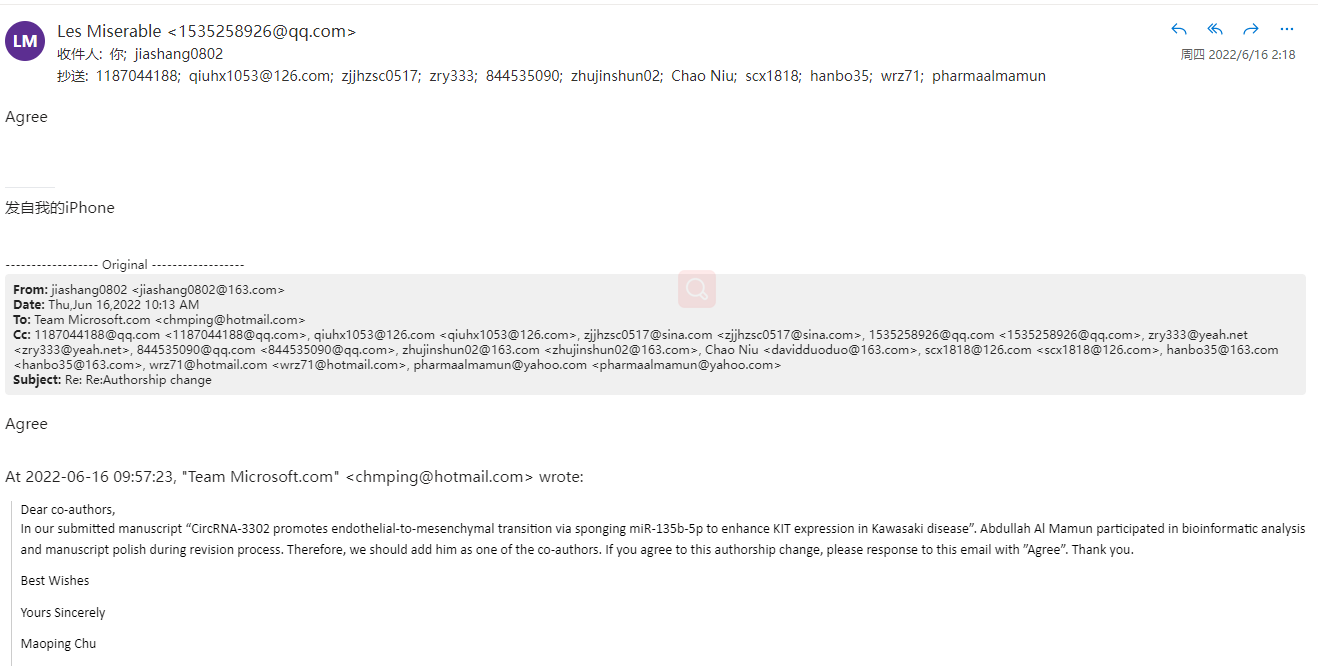


**Ruiyin Zhang**


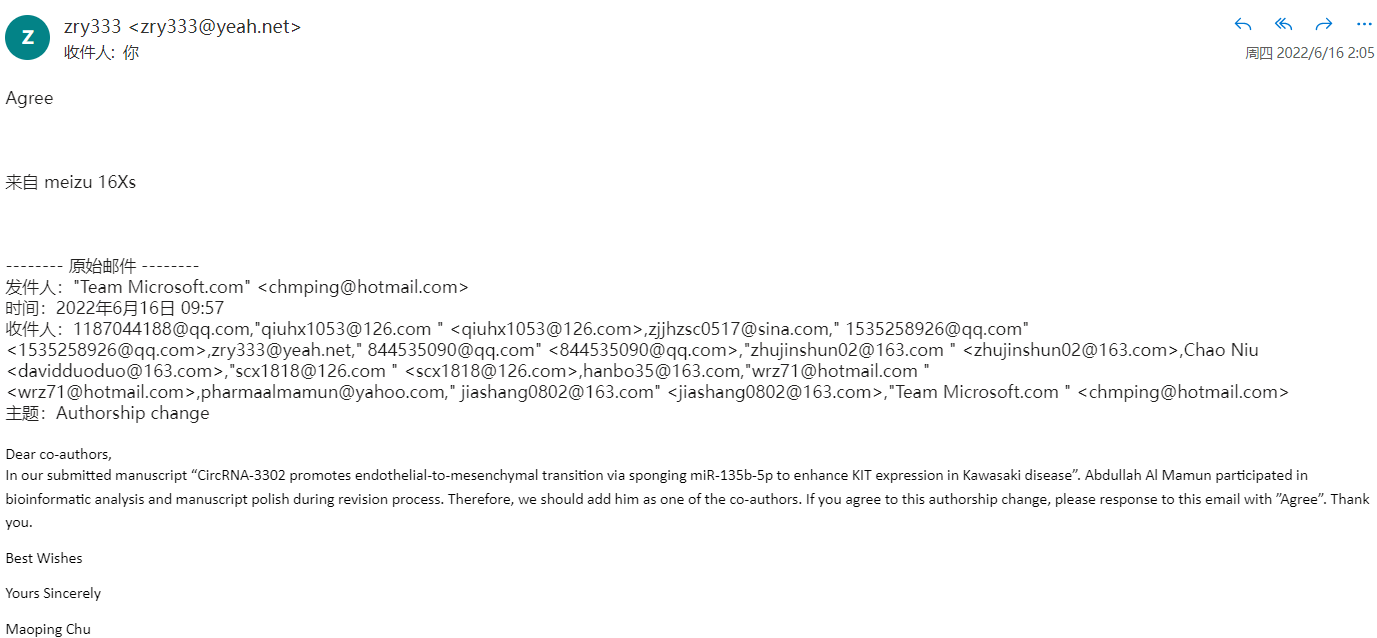


**Jinhui Zhou**


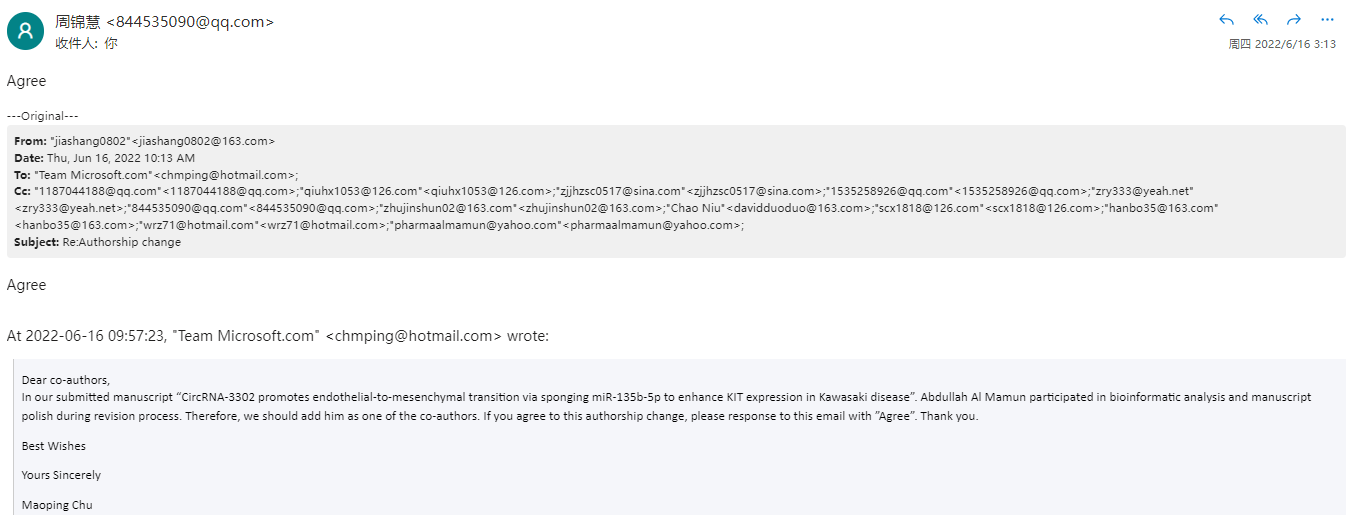


**Jinshun Zhu**


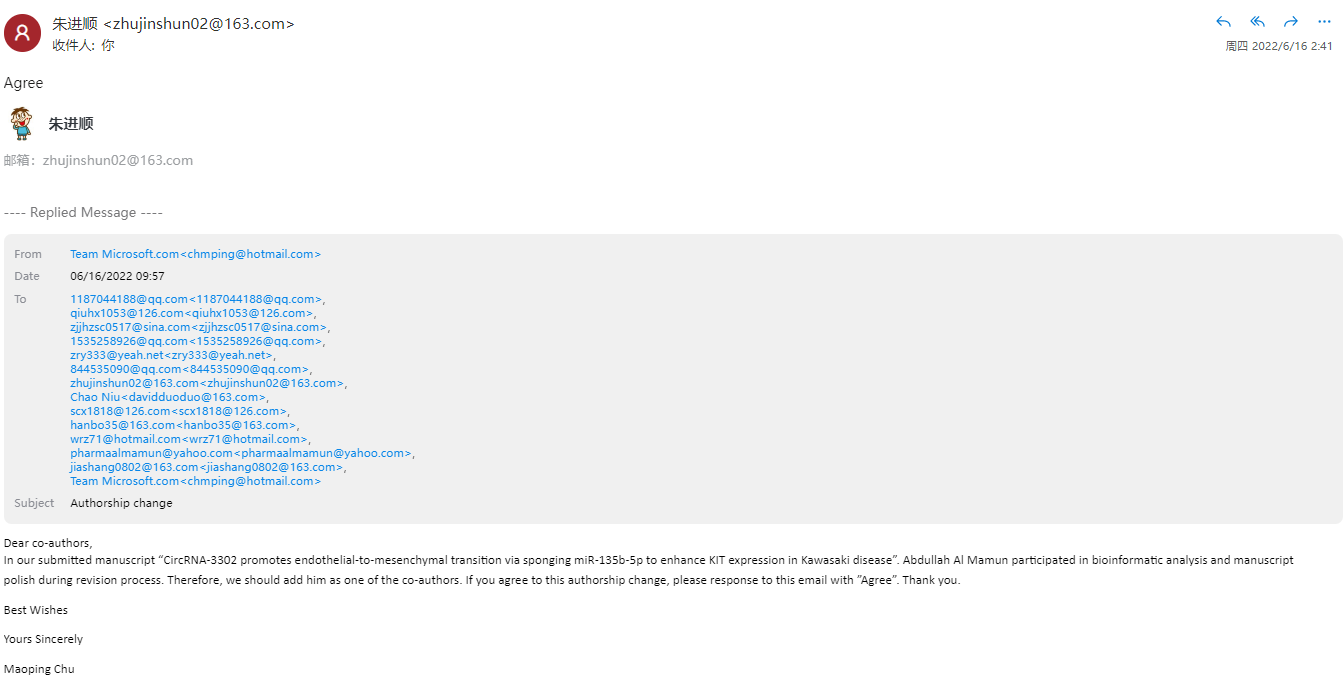


**Chao Niu**


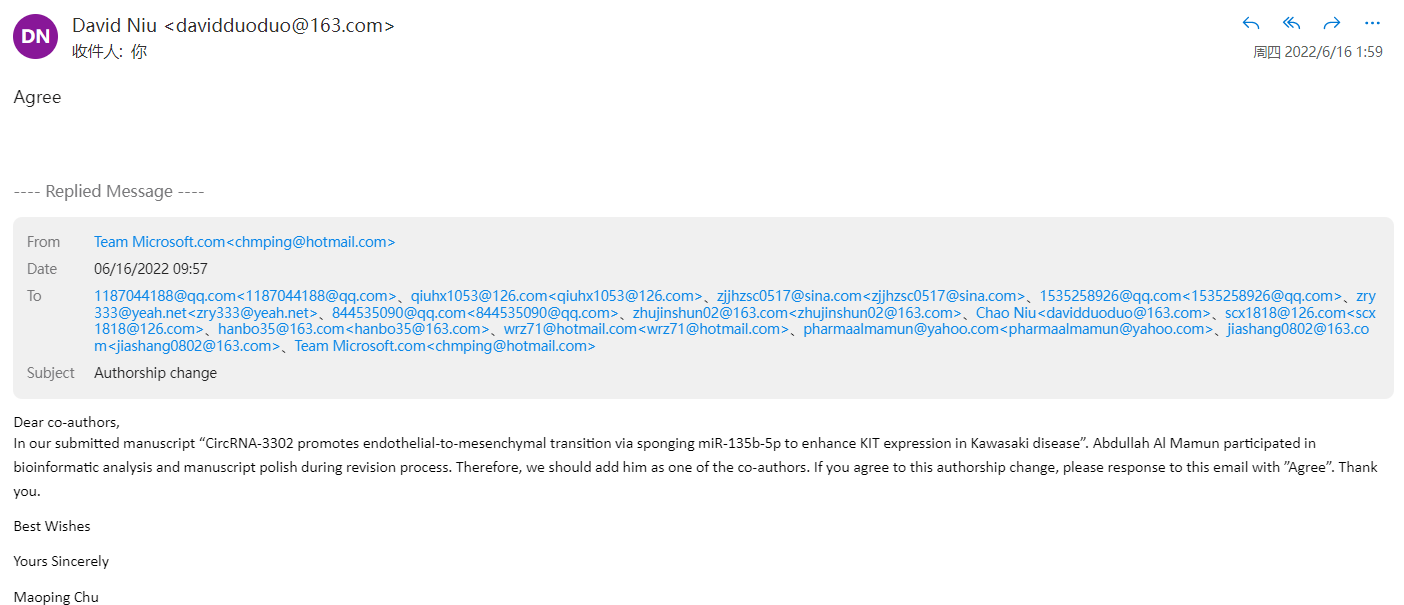


**Rongzhou Wu**


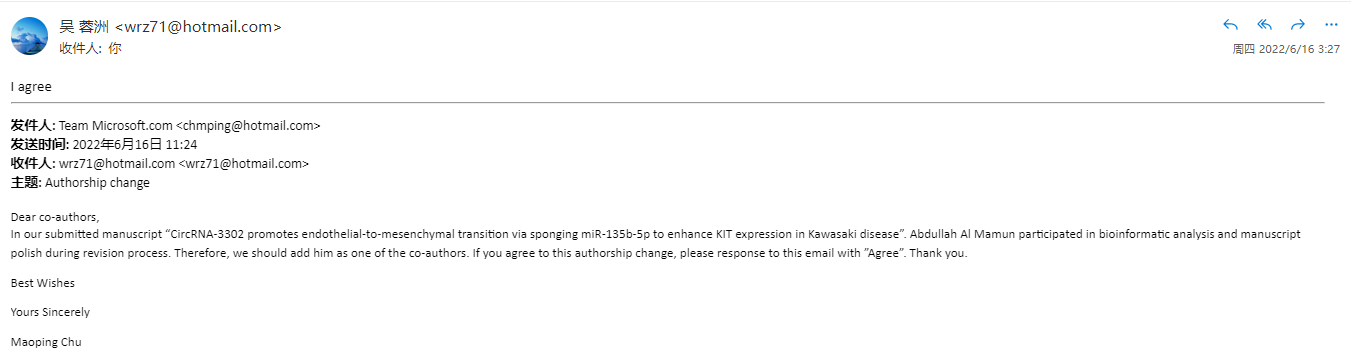


**Chuxiao Shao**


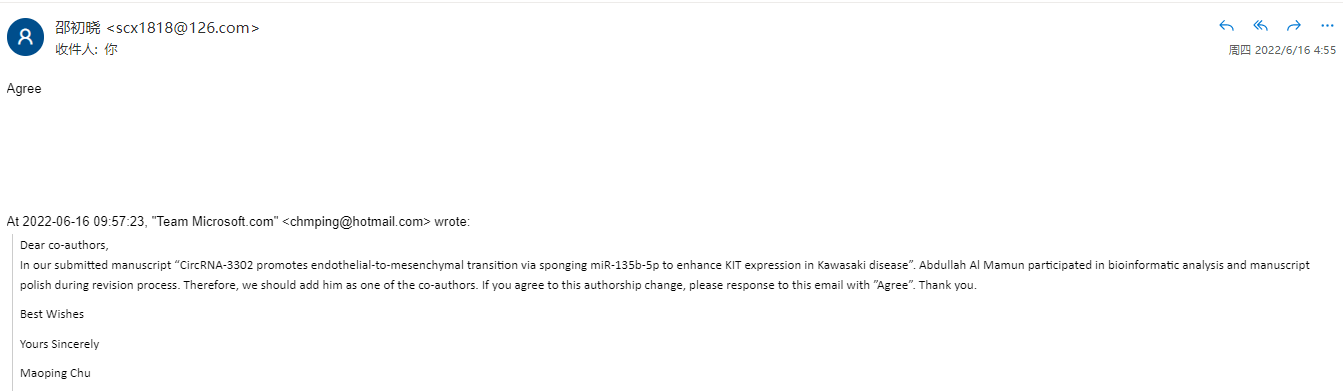


**Abdullah Al Mamun**


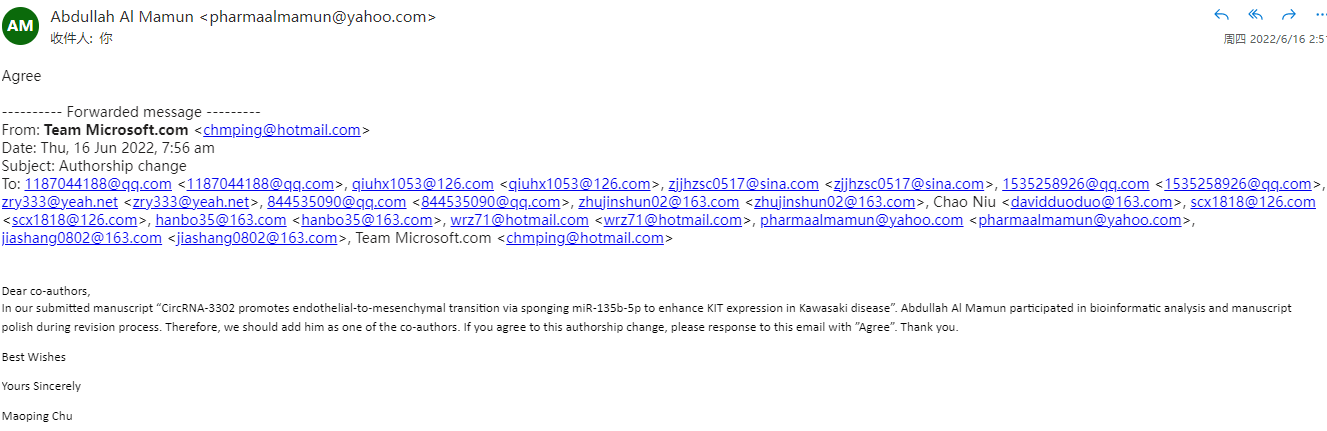


**Bo Han**


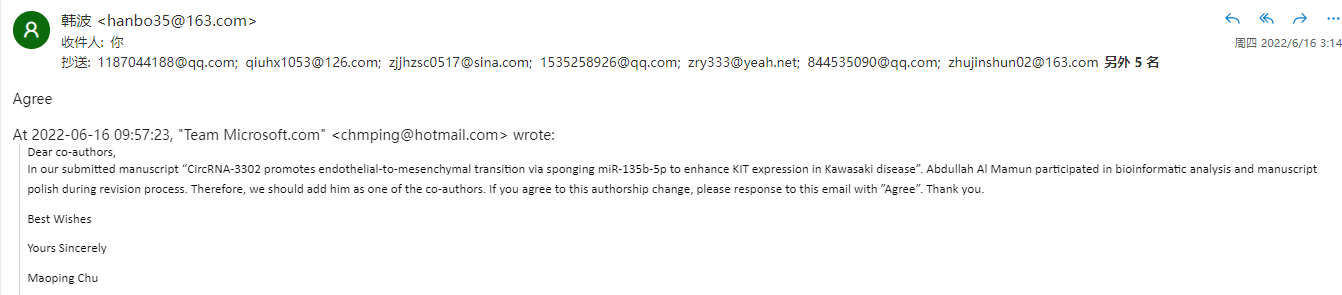


**Maoping Chu**


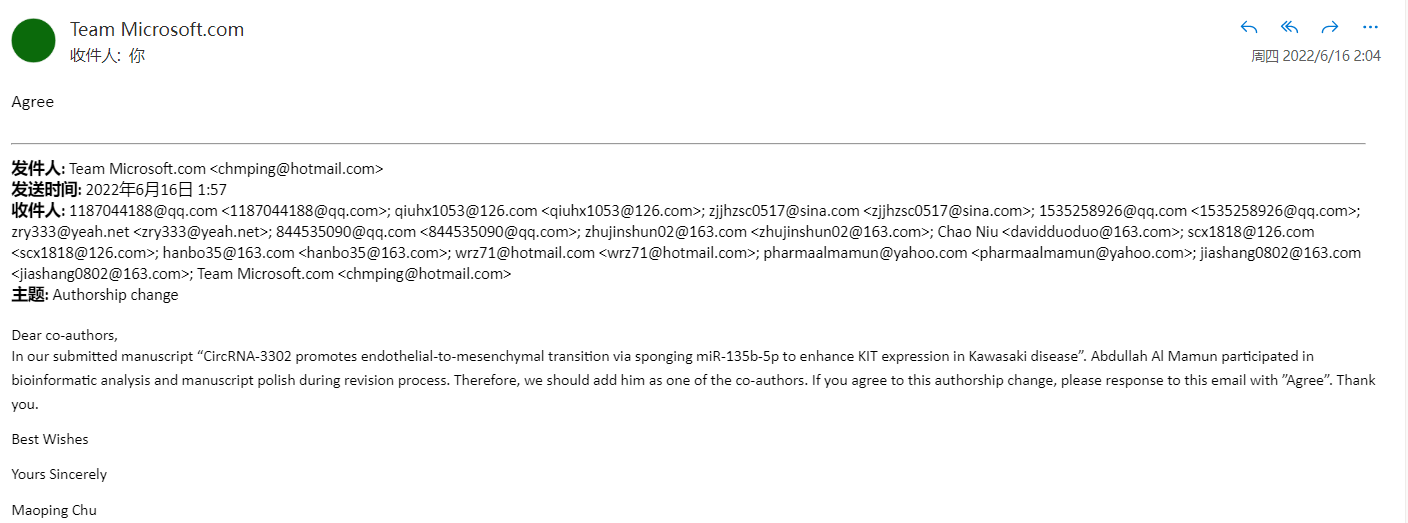


**\**

**Chang Jia**


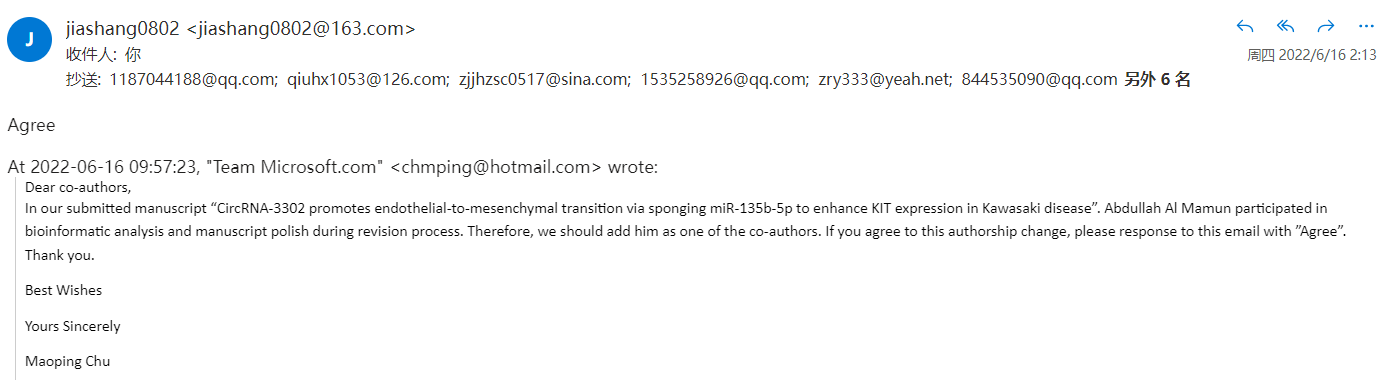

Supplement: Supplementary file 3 — Agreement with the change of authorship [file 41420_2022_1092_MOESM3_ESM.doc]
